# Automatic Optimization of an *in Silico* Model of Human iPSC Derived Cardiomyocytes Recapitulating Calcium Handling Abnormalities

**DOI:** 10.3389/fphys.2018.00709

**Published:** 2018-06-26

**Authors:** Michelangelo Paci, Risto-Pekka Pölönen, Dario Cori, Kirsi Penttinen, Katriina Aalto-Setälä, Stefano Severi, Jari Hyttinen

**Affiliations:** ^1^Faculty of Biomedical Sciences and Engineering, BioMediTech Institute, Tampere University of Technology, Tampere, Finland; ^2^Faculty of Medicine and Life Sciences, BioMediTech Institute, University of Tampere, Tampere, Finland; ^3^Department of Electrical, Electronic and Information Engineering “Guglielmo Marconi”, University of Bologna, Cesena, Italy; ^4^Heart Hospital, Tampere University Hospital, Tampere, Finland

**Keywords:** human induced pluripotent stem cell-derived cardiomyocyte, action potential, calcium transient, computer simulation, *in silico* modeling

## Abstract

The growing importance of human induced pluripotent stem cell-derived cardiomyoyctes (hiPSC-CMs), as patient-specific and disease-specific models for studying cellular cardiac electrophysiology or for preliminary cardiotoxicity tests, generated better understanding of hiPSC-CM biophysical mechanisms and great amount of action potential and calcium transient data. In this paper, we propose a new hiPSC-CM *in silico* model, with particular attention to Ca^2+^ handling. We used (i) the hiPSC-CM Paci2013 model as starting point, (ii) a new dataset of Ca^2+^ transient measurements to tune the parameters of the inward and outward Ca^2+^ fluxes of sarcoplasmic reticulum, and (iii) an automatic parameter optimization to fit action potentials and Ca^2+^ transients. The Paci2018 model simulates, together with the typical hiPSC-CM spontaneous action potentials, more refined Ca^2+^ transients and delayed afterdepolarizations-like abnormalities, which the old Paci2013 was not able to predict due to its mathematical formulation. The Paci2018 model was validated against (i) the same current blocking experiments used to validate the Paci2013 model, and (ii) recently published data about effects of different extracellular ionic concentrations. In conclusion, we present a new and more versatile *in silico* model, which will provide a platform for modeling the effects of drugs or mutations that affect Ca^2+^ handling in hiPSC-CMs.

## Introduction

Human induced pluripotent stem cell-derived cardiomyocytes (hiPSC-CMs) are cardiac cells derived from stem cells, which have been produced by donor's differentiated cells by means of reprogramming (Takahashi et al., 2007). The role of hiPSC-CMs, has become more and more pervasive in basic electrophysiological studies as well as in applied research, such as pharmacological tests, since their discover in 2007. As an *in vitro* human model, hiPSC-CMs strongly impacted the study of biophysical mechanisms underlying cardiac electrophysiology at cellular level, both in control and diseased conditions. Especially hiPSC-CMs' patient- and disease-specificity is fundamental to assess the effects of genetic mutations, such as Long QT (LQT) (Moretti et al., 2010; Lahti et al., 2012; Ma et al., 2013), catecholaminergic polymorphic ventricular tachycardia (CPVT) (Kujala et al., 2012) and hypertrophic cardiomyopathy (HCM) (Ojala et al., 2016), on the functionality of cardiomyocytes. Ever since the beginning of the Comprehensive *In vitro* Proarrhythmic Assay (CIPA) (http://cipaproject.org/) in 2013, hiPSC-CMs have had a dramatic impact on pharmacology, serving as a powerful *in vitro* model to test the *in silico* model predictions regarding cardiac safety or drug toxicity at cellular level.

During the last 10 years, many progresses were done in terms of efficiency of hiPSC-CM production and availability of commercial cell lines. This enabled also the hiPSC-CM electrophysiological and pharmacological evaluation by means of medium-throughput (Rajamohan et al., 2016) or even high-throughput systems (Entcheva and Bub, 2016; Klimas et al., 2016), where the use of voltage- and calcium-sensitive dyes has been combined with hiPSC-CM optogenetic stimulation. Together with the availability of these experimental data, new methods have been developed to process them (Björk et al., 2017; Ahola et al., 2018).

The importance of Ca^2+^ cycling to basic cardiac functionality, together with the growing availability of hiPSC-CM Ca^2+^ cycling data, makes Ca^2+^ transients, and biomarkers computed onto them, as interesting as action potential (AP) measurements. Ca^2+^ is fundamental in the heart excitation-contraction (EC) coupling, i.e., how the electrical and the mechanical properties of the heart are linked together and how the AP leads to the cardiomyocyte contraction. The elements involved in this phenomenon are the L-type Ca^2+^ channels, the sarcoplasmic reticulum (SR) and the sarcomeres, i.e., the contractile unit of the cardiomyocyte. During the AP upstroke, the L-type Ca^2+^ channels open and Ca^2+^ flows into the cardiomyocyte. This Ca^2+^ influx is sufficient to trigger the Ca^2+^ release from SR through the ryanodine-sensitive Ca^2+^ channels, which increases the cytosolic Ca^2+^. Such amount of Ca^2+^ allows starting the crossbridge cycle, which is at the basis of the cardiomyocite contraction and continues until Ca^2+^ is restored to its basal cytosolic concentration. This is mainly done by the SERCA-2 pump, which reabsorbs Ca^2+^ from the cytosol into the SR. Moreover, cytosolic Ca^2+^ is extruded into the extracellular space by the Na^+^/Ca^2+^ exchanger (I_NaCa_) and by the sarcolemmal Ca^2+^ pump (I_pCa_). All these mechanisms make the intracellular Ca^2+^ concentration change, thus producing Ca^2+^ transients associated to the APs (Walker and Spinale, 1999).

In 2013 we published the first *in silico* hiPSC-CMs model (Paci et al., 2013), based on our previous model of cardiomyocytes derived from human embryonic stem cells (Paci et al., 2012) and on the experimental data by Ma et al. (2011). This model has been widely used for computational studies, such as (i) the prediction of drug effects on cardiac electrophysiology (Paci et al., 2015; Lei et al., 2017), (ii) the model extension to multielectrode array simulations (Raphel et al., 2017), and (iii) the assessment of hiPSC-CM electrophysiological variability in control and mutant cells, by means of populations of *in silico* hiPSC-CMs (Paci et al., 2017). However, one of the Paci2013 model limitations resides in its formulations of the Ca^2+^ handling system: especially the Ca^2+^ release from the sarcoplasmic reticulum (SR) is formulated with the functional but quite elementary Ca^2+^ release from the TenTusscher2004 model (ten Tusscher et al., 2004).

In this work, we propose an updated version of the Paci2013 hiPSC-CM ventricular-like model with a more flexible Ca^2+^ handling formulation. The Ca^2+^ transients produced by the model were calibrated on experimental Ca^2+^ imaging data recorded in our laboratory on hiPSC-CMs. Moreover, the fine tuning of the model parameters was performed by means of an automatic optimization technique (Fabbri et al., 2017), in order to reproduce realistic AP and Ca^2+^ transient shapes and to speed up the parameter tuning phase. Parameter optimization affected only the parameters representing the Ca^2+^ SR fluxes and a very limited set of parameters of membrane currents in order to be consistent with the voltage clamp experiment fitting done by Paci et al. (Paci et al., 2013). Finally, the resulting updated model was validated against ion current blocking data.

## Materials and methods

### New Ca^2+^ handling system formulation

The main limitation of the original Paci2013 model (Paci et al., 2013) and its following minor updates (Paci et al., 2015, 2017) is the simplified description of the Ca^2+^ release from SR, i.e., the release current I_rel_. I_rel_ was formulated as in the TenTusscher2004 model (ten Tusscher et al., 2004) in the following way:

$$\begin{array}{l} I_{rel}=K_{rel}\cdot \left(c_{rel}+\frac{a_{rel}\cdot Ca_{SR}^{2}}{b_{rel}^{2}+Ca_{SR}^{2}}\right)\cdot d\cdot g \end{array}$$

where *K*_*rel*_, *a*_*rel*_, *b*_*rel*_, and *c*_*rel*_ are constants, Ca_SR_ is the Ca^2+^ concentration in the SR reticulum, d is the L-type Ca^2+^ current (I_CaL_) voltage-dependent activation gate, and g is the *I*_*rel*_ specific inactivation gate. Therefore, I_rel_ is not activated by the cytosolic Ca^2+^ concentration Ca_i_ sensed by the Ryanodine-sensitive receptor located on the SR membrane, but it is triggered the same way as I_CaL_. Due to this mechanism, the model was not able to produce proarrhythmic triggers, such as delayed afterdepolarization (DADs) (Fink et al., 2011).

We reformulated *I*_*rel*_ according to the formulation used by Koivumäki et al. (2011) for the human atrial myocyte:

$$\begin{array}{l} I_{rel}=I_{rel,max}\cdot RyR_{CaSR}\cdot RyR_{o}\cdot RyR_{c}\cdot \left(Ca_{SR}-Ca_{i}\right) \end{array}$$

where I_rel,max_ represents the maximum Ca^2+^ release from SR, *RyR*_*CaSR*_ is the dependence on *Ca*_*SR*_, *RyR*_*o*_ is the open (activation) gating variable, *RyR*_*c*_ the closed (inactivation) gating variable. A third gating variable, *RyR*_*a*_, was used to modulate the working point (adaptation) of the *RyR*_*o*_ and *RyR*_*c*_ gates according to the cytosolic Ca^2+^ concentration as in Koivumäki et al. (2011). The formulation of the single gating variables is the following

$$\begin{array}{lll} RyR_{CaSR} & = & 1-\frac{1}{1+e^{\frac{Ca_{SR}-0.3}{0.1}}}\\ RyR_{a,ss} & = & RyR_{a1}-\frac{RyR_{a2}}{1+e^{\frac{Ca_{i}-RyR_{a,half}}{RyR_{a,k}}}}\\ RyR_{o,ss} & = & 1-\frac{1}{1+e^{\frac{Ca_{i}-\left(RyR_{a}+RyR_{o,half}\right)}{RyR_{o,k}}}}\\ RyR_{c,ss} & = & \frac{1}{1+e^{\frac{Ca_{i}-\left(RyR_{a}+RyR_{c,half}\right)}{RyR_{c,k}}}} \end{array}$$

where the subscript ss indicates the steady state value of the gating variable and the subscripts half and k indicate the half Ca^2+^ concentration and the slope, respectively, of the gating variable steady state. The values of the constants *RyR*_*a*1_, *RyR*_*a*2_ and the gating variable half activations *RyR*_*a,half*_, *RyR*_*o,half*_ and *RyR*_*c,half*_ were then optimized (together with other parameters) as described in section “Parameter Optimization.” We report the full set of equations in section 1 of the Supplementary Material.

The formulation of the other SR fluxes, namely the SERCA pump (I_up_) and the leakage current (I_leak_) were not changed with respect to the Paci2013 model (Paci et al., 2013). However, their parameters went through the optimization process, as we describe in section Parameter Optimization. In the following we will refer as Paci2013 to the model version presented in Paci et al. (2017).

### Parameter optimization

The parameter optimization process was adapted from Fabbri et al. (2017).

Shortly, parameter optimization was done using the Matlab© function *fminsearch*, which implements the Nelder-Mead Simplex Method, as reported in Fabbri et al. (2017). Such function minimizes a cost function built on the experimental biomarkers we want the model to simulate. The following equations show the cost function structure (Fabbri et al., 2017):

$$\begin{array}{l} Cost=\sum_{i\;=\;1}^{N_{biomarkers}}Cost_{i}\\ Cost_{i}=\{\begin{array}{ll} \frac{\left|b_{i,Exp}-b_{i,Sim}\right|-SD_{i}}{SD_{i}}\cdot w_{i} & if\left|b_{i,Exp}-b_{i,Sim}\right|>SD\\ 0 & otherwise \end{array} \end{array}$$

where *Cost*_*i*_ and *SD*_*i*_ represent the cost and the standard deviation for a single biomarker b_i_.

The structure of the cost function Cost is consistent with the cost function used in Fabbri et al. (2017). The contribution of each biomarker to the overall cost is zero if the biomarker is within its ±SD range, otherwise it grows linearly. Moreover, each contribution *Cost*_*i*_ was weighted according to the respective weight *w*_*i*_. This results in a non-linear cost function, which is zero if all the biomarkers are within the experimental ranges and greater than zero if at least one biomarker is out of range. The AP experimental biomarkers considered for the cost function were: AP amplitude (APA), maximum diastolic potential (MDP), cycle length (CL), maximum upstroke velocity (V_max_), AP duration at 10, 30, and 90% of repolarization (APD_10_, APD_30_, APD_90_) and AP shape factor (Triangulation). Triangulation is a shape factor used by Ma et al. (2011) to discriminate between atrial-like (Triangulation < 1.5) and ventricular-like (Triangulation>1.5) APs, and it is computed as:

$$\begin{array}{l} Triangulation=\frac{APD_{30}-APD_{40}}{APD_{70}-APD_{80}} \end{array}$$

The Ca^2+^ transient experimental biomarkers were: duration of the Ca^2+^ transient (DURATION), time to peak (TPEAK), rise time from 10 to 50% (RT_1050_), rise time from 10 to 90% (RT_1090_), decay time from 90 to 10% (DT_9010_) and the Ca^2+^ transient rate (FREQ). The full list of the parameters optimized by this method and their original values are reported in Table 1. For optimization, we only chose parameters related to Ca^2+^ handling and additional parameters of I_NaCa_ and the Na^+^/K^+^ pump (I_NaK_), i.e., those currents for which the Ma et al. dataset (Ma et al., 2011) did not provide experimental data. The parameter values were constrained in a range [−20%, +20%] with respect to their nominal value in the Paci2013 model, in order to avoid non-physiological values, such as negative conductances. The initial parameter values for RyR_a1_, RyR_a2_, RyR_a,half_, RyR_o,half_, RyR_c,half_ were rescaled to 1/10 of their values in Koivumäki et al. (2011) before the optimization procedure, in order to adapt them to the Ca^2+^ concentrations in the Paci2013 model, and then optimized as the other parameters. All the other ionic current parameters were kept as in Paci et al. (2013) and Paci et al. (2017) for the late Na^+^ current (I_NaL_) only.

**Table 1:** Parameters chosen for optimization.

**Parameter**	**Description**	**Original value**	**Optimized value**
V_max,up_ (mM/s)	Maximum Ca^2+^ SERCA uptake	0.56064	0.5113
I_rel,max_ (mM/s)	Maximum Ca^2+^ release current	—	62.5434
RyR_a1_ (μM)	Adaptation gate constant 1	—	0.05354
RyR_a2_ (μM)	Adaptation gate constant 2	—	0.0488
RyR_a,half_ (μM)	Activation gate half Ca^2+^ concentration	—	0.02427
RyR_o,half_ (μM)	Open gate half Ca^2+^ concentration	—	0.01042
RyR_c,half_ (μM)	Close gate half Ca^2+^ concentration	—	0.00144
I_NaCa,max_ (pA/pF)	Maximum Na^+^/Ca^2+^ exchange current	5.978e3	3917.0463
I_NaK,max_ (pA/pF)	Maximum Na^+^/K^+^ pump current	2.2958	2.6351
K_up_ (mM)	SERCA half saturation constant	2.5e-4	3.1928e-4
I_leak,max_ (1/s)	Maximum Ca^2+^ leakage flux from SR	4.444e-4	4.7279e-4
alpha (-)	Factor enhancing outward nature of I_NaCa_	2.8571432	2.5371

*For each parameter we reported its original value as in Paci et al. (2017) and the new optimized value. We marked those parameters, which were not included in the old version of the model, as —*.

To constrain the model parameters, two different experimental datasets were used. AP data were taken from Ma et al. (2011). This is the same dataset used to calibrate the Paci2013 model and it includes biomarkers computed on spontaneous ventricular-like hiPSC-CM APs recorded at 35–37°C. A second, new, dataset was obtained through Ca^2+^ transient recordings performed at the BioMediTech Institute (Tampere, Finland).

### Calcium recordings in hiPSC-CMs

This study was carried out in accordance with the recommendations of Guidelines of the Ethics Committee of Pirkanmaa Hospital District (Tampere, Finland). The protocol was approved by the Ethics Committee of Pirkanmaa Hospital District (Aalto-Setälä R08070). All subjects gave written informed consent in accordance with the Declaration of Helsinki. New Ca^2+^ transient dataset were recorded at the BioMediTech Institute from healthy control hiPSC-CMs at 35–37°C. The generation and characterization of the control hiPSC line and cardiac differentiation were done as described earlier (Ojala et al., 2016). Cardiomyocytes plated on a coverslip were loaded with 4 μM Fluo-4 AM (Thermo Fisher Scientific) for 30 min and de-esterified for 10 min in perfusate medium: (in mM) 137 NaCl, 5 KCl, 0.44 KH_2_PO_4_, 20 HEPES, 4.2 NaHCO_3_, 5 D-glucose, 2 CaCl_2_, 1.2 MgCl_2_, and 1 Na-pyruvate dissolved in H_2_O (all from Sigma Aldrich). pH of the perfusate medium was adjusted to 7.4 with NaOH (Sigma Aldrich). Perfusate was heated with an inline heater SH-27B controlled with a TC-324B controller unit and input into a RC-25 imaging chamber (all Warner instruments Inc., CT, USA). Calcium kinetics of spontaneously beating cardiomyocytes were imaged with an inverted Olympus IX70 microscope using UApo/340 0,75NA 20x air objective (Olympus, Tokyo, Japan) and recorded with ANDOR iXon 885 EM-CCD camera (Andor Technology, Belfast, Northern Ireland) using 2 × 2 binning and synchronized with a Polychrome V light source by a real time DPS control unit. LiveAcquisition software (TILL Photonics, Munich, Germany) was used to control light source and camera during recording. Fluo-4 was excited at 490 nm wavelength and the emission was recorded through Olympus U-MF2 Alexa 488 band-pass filter cube (ex.470–495, em.525/50 nm).

Data were recorded from 15 cells for a total of 218 transients. For each biomarker mean value and standard deviation (SD) were computed for model calibration. The experimental values for the biomarkers and their weights are reported in Table 2.

**Table 2:** Experimental and simulated values of the biomarkers considered for the parameter optimization.

**Biomarker**	**Weight in the cost function**	**Experimental value (mean ± SD)**	**Simulated value**
APA (mV)	1	104 ± 6.0	109.6
MDP (mV)	2	−75.6 ± 6.6	−75.8
CL (ms)	2	1700.0 ± 547.7	1549.3
V_max_ (V/s)	1	27.8 ± 26.3	24.8
APD_10_ (ms)	1	74.1 ± 26.3	85.8
APD_30_ (ms)	1	180 ± 58.6	241.8
APD_90_ (ms)	1	414.7 ± 119.4	379.8
Triangulation (-)	1	2.5 ± 1.1	3.0
DURATION (ms)	1	804.5 ± 188.0	617.8
TPEAK (ms)	1	270.4 ± 108.3	165.9
RT_1050_ (ms)	1	82.9 ± 50.5	47.7
RT_1090_ (ms)	1	167.3 ± 69.8	106.0
DT_9010_ (ms)	1	409.8 ± 100.1	367.6
FREQ (Hz)	1	0.70 ± 0.38	0.65

*Comparison between the simulated biomarkers and the experimental ones used for the model parameter optimization. AP biomarkers: AP amplitude (APA), maximum diastolic potential (MDP), cycle length (CL), maximum upstroke velocity (V_max_), AP duration at 10, 30, and 90% of repolarization (APD_10_, APD_30_, APD_90_) and AP shape factor (Triangulation). Ca^2+^ transient biomarkers: duration of the transient (DURATION), time to peak (TPEAK), rise time from 10 to 50% and to 90% (RT_1050_, RT_1090_), decay time from 90 to 10% (DT_9010_) and the Ca^2+^ transient rate (FREQ)*.

### Model validation with current blocker simulations

In order to validate the new hiPSC-CM model after the introduction of the new I_rel_ formulation and the parameters optimization by means of our Ca^2+^ transient data, we chose to replicate the current blocker simulations performed in Paci et al. (2013). The model was paced at 1 Hz for 800 s to reach its steady state, then the current blocker was simulated by reducing the maximum conductance of the affected current and finally the AP biomarkers were computed after 400 s. The current blockers considered in the simulations were the same used by Ma et al. (2011): tetrodotoxine (TTX, I_Na_ blocker), nifedipine (NIFED, I_CaL_ blocker), E4031 (I_Kr_ blocker) and 3R4S-Chromanol 293B (CHR, I_Ks_ blocker). For TTX, NIFED and E4031 we considered the following IC_50_ values: 0.64 μM (Ma et al., 2011), 0.038 μM (Ma et al., 2011) and 100 nM (Sanguinetti and Jurkiewicz, 1990; Gerlach et al., 2010) respectively. In Ma et al. (2011) CHR had small effects on the AP biomarkers, therefore we tested a range of block levels: 30, 50, 70, and 90%. The stimulus current was 550 pA for NIFED, E4031 and CHR, while we chose 750 pA for TTX in order to trigger APs also at the highest blocker concentrations.

### Delayed afterdepolarizations

In order to trigger DADs in spontaneous APs, Ca^2+^ overload had to be simulated: we chose to increase the superfusate Ca^2+^ concentration (Volders et al., 2000). To ensure that Ca^2+^ overload was simulated only once the model was in steady state, we ran a 800 s simulation without external pacing. From these steady state conditions, we increased the extracellular Ca^2+^ concentration by setting it to 3.945 mM and again we simulated the spontaneous APs. We ran this protocol with both the Paci2013 and the Paci2018 models.

## Results

### Spontaneous action potentials and Ca^2+^ transients

**Figure 1:**
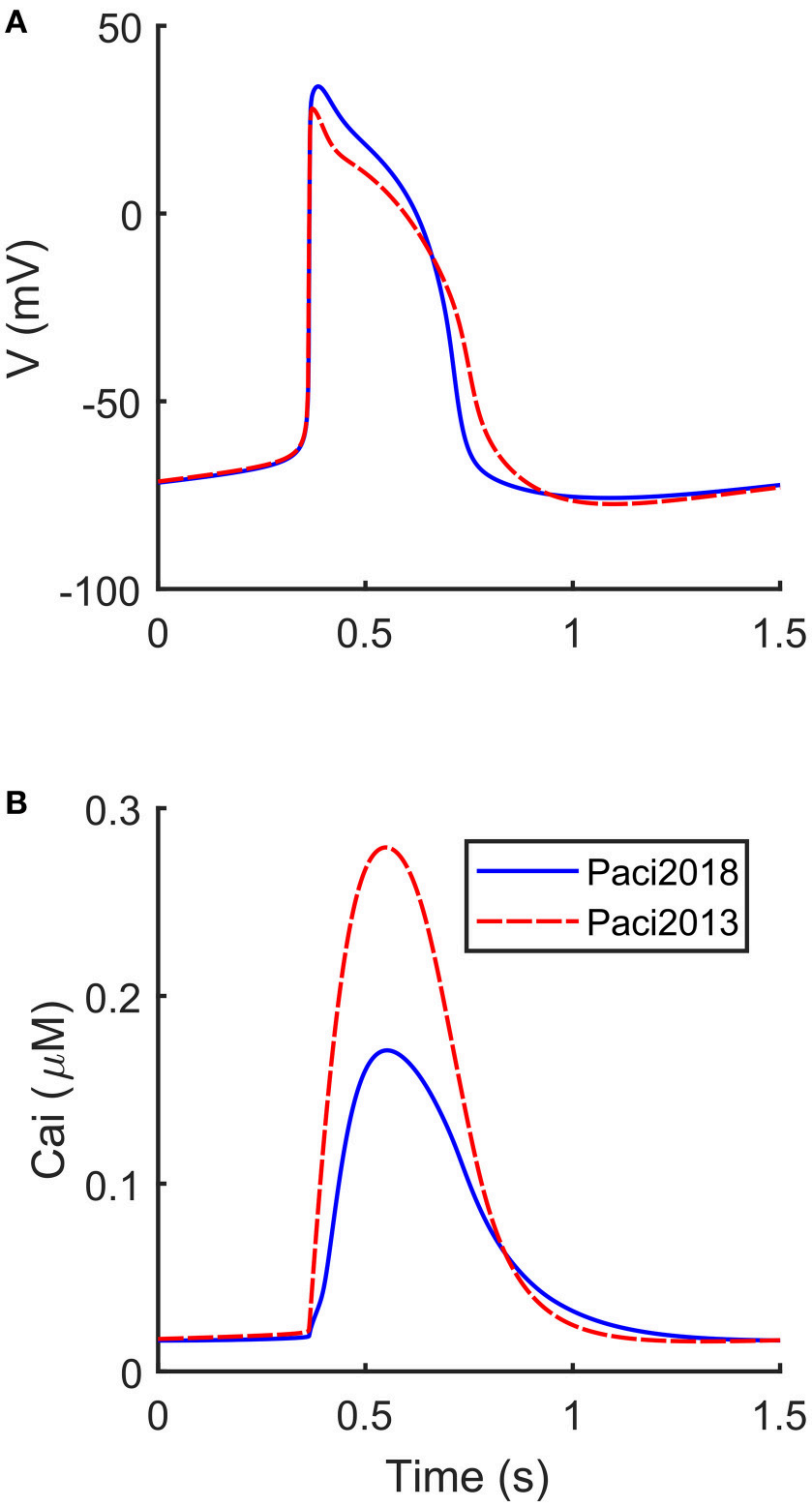
Comparison between the APs and Ca^2+^ transients simulated by the new Paci2018 model (solid blue) and the Paci2013 model (dashed red). **(A)** membrane potential. **(B)** Ca^2+^ transient.

**Figure 2:**
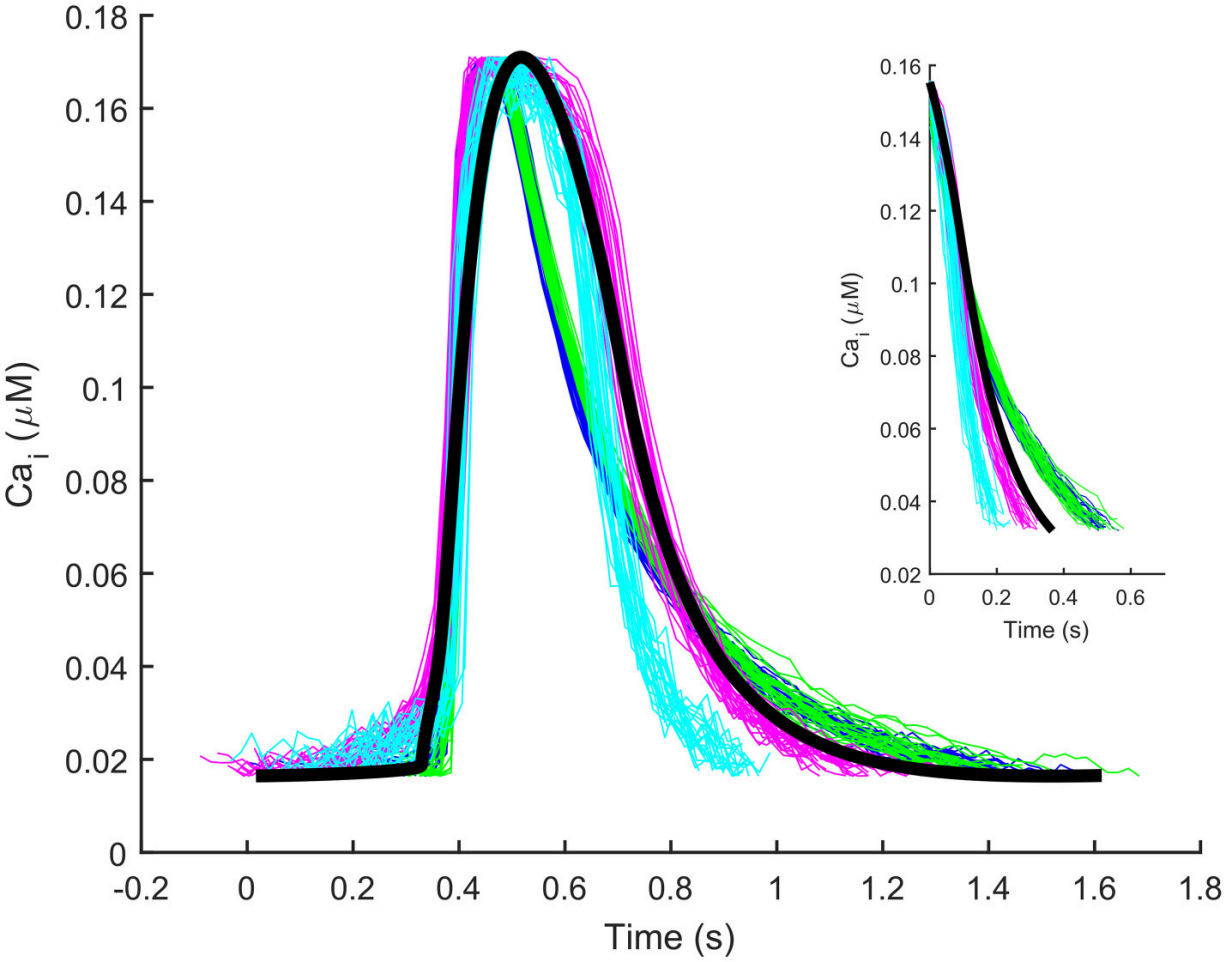
Illustrative experimental Ca^2+^ transients from four cells (blue, cyan, green, magenta) and the Ca^2+^ transient simulated by the Paci2018 model (black). The y-axis of the experimental traces (originally ΔF/F) was normalized between the minimum and maximum values of the simulated trace for comparison. The inset contains only the decay part (from 90 to 10% of the transient amplitude) of the experimental and simulated transients reported in the main Figure.

**Figure 3:**
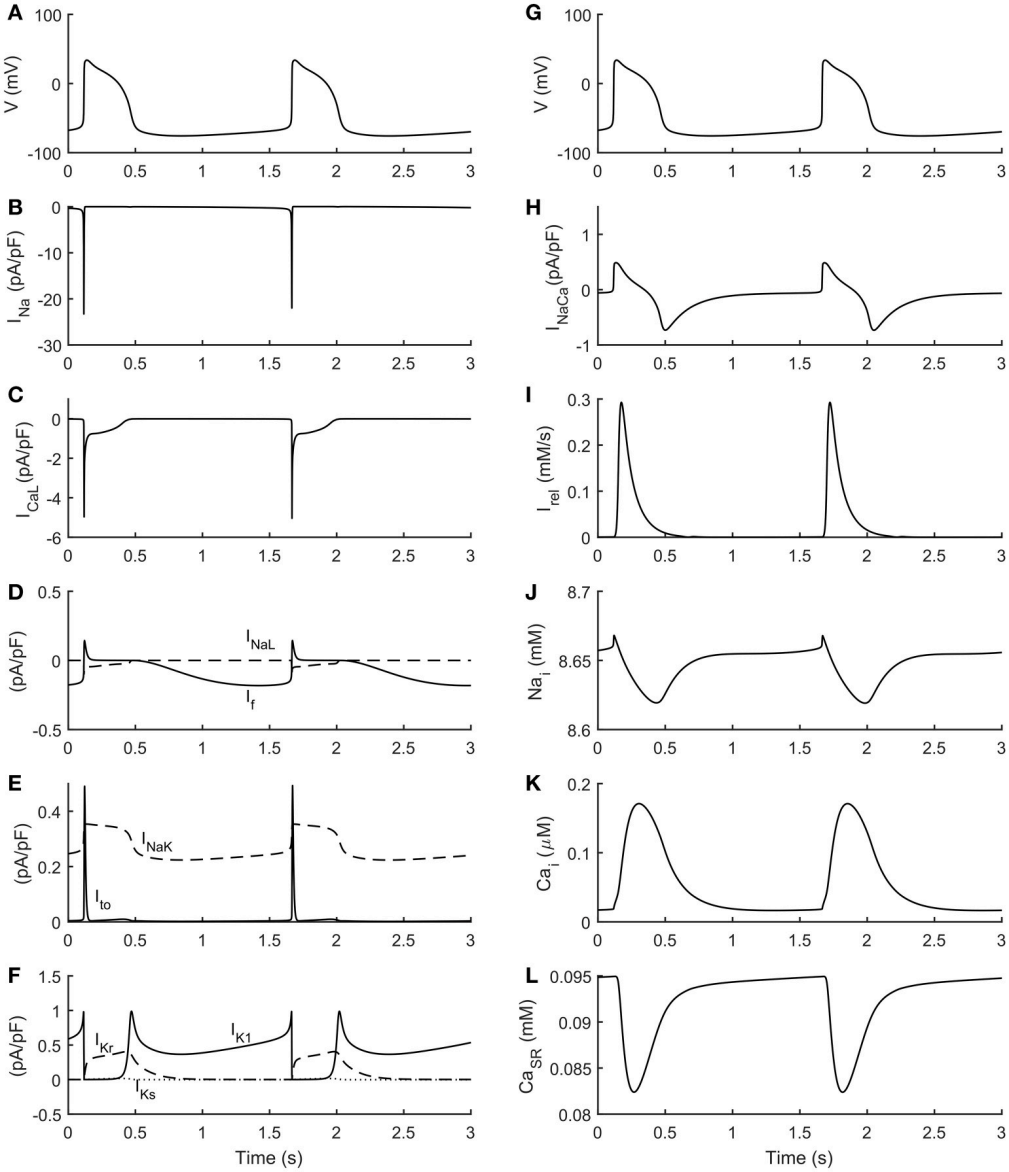
Simulated spontaneous action potentials and ionic currents. **(A,G)** membrane potential. **(B)** Fast Na^+^ current (I_Na_). **(C)** L-type Ca^2+^ current (I_CaL_). **(D)** Funny current (I_f_) and Late Na^+^ current (I_NaL_). **(E)** Transient outward K^+^ current (I_to_) and Na^+^/K^+^ pump (I_NaK_). **(F)** Rapid delayed (I_Kr_), slow delayed (I_Ks_) and inward (I_K1_) rectifier K^+^ currents. **(H)** Na^+^/Ca^2+^ exchanger (I_NaCa_). **(I)** Release current from sarcoplasmic reticulum (I_rel_). **(J)** Na^+^ cytosolic concentration (Na_i_). **(K)** Cytosolic Ca^2+^ concentration (Ca_i_). **(L)** Sarcoplasmic Ca^2+^ concentration (Ca_SR_).

**Figure 4:**
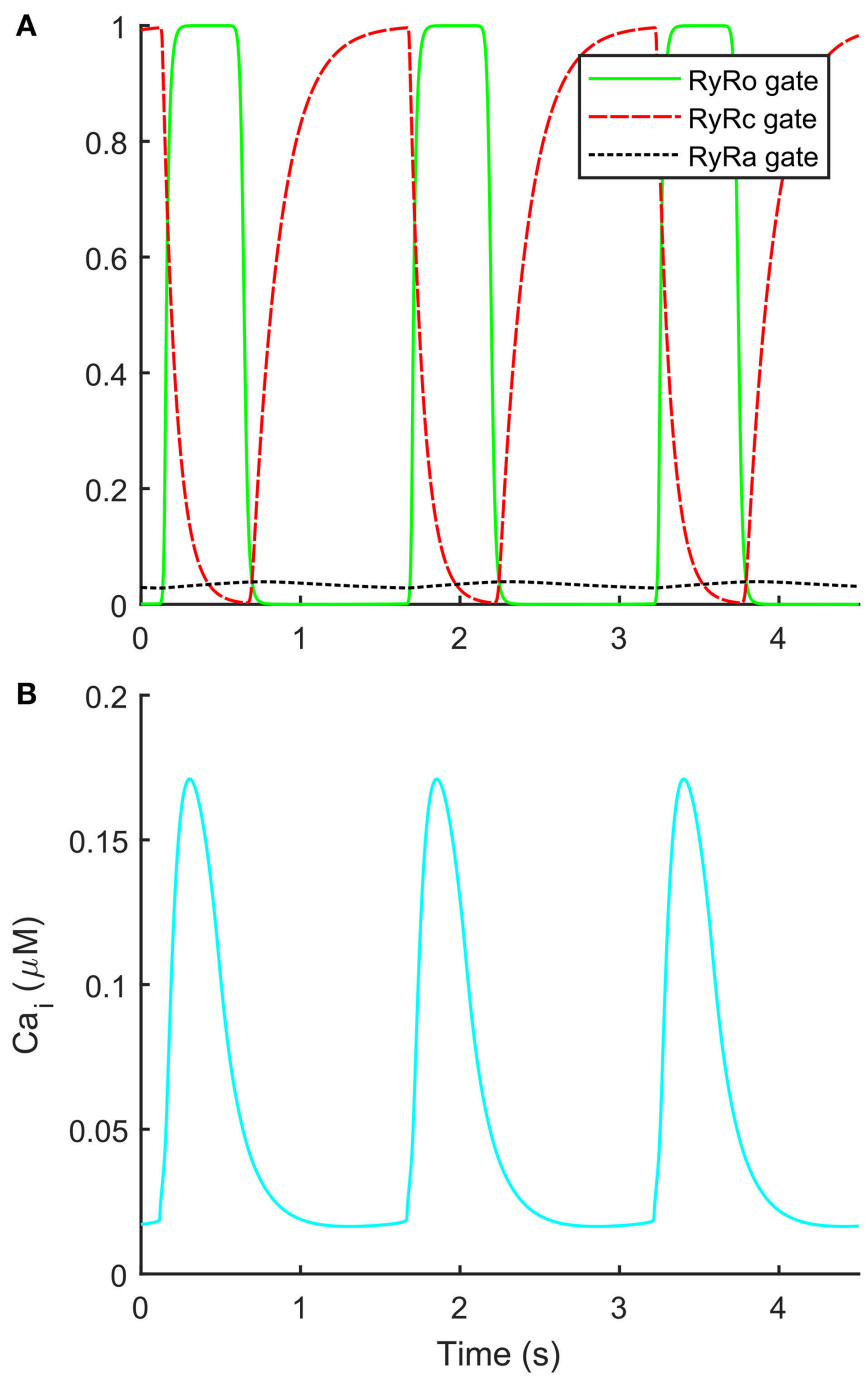
Details on the new formulation of the Ca^2+^ release from sarcoplasmic reticulum. **(A)** Time-course of the three sarcoplasmic release current gates during spontaneous action potentials. **(B)** Ca^2+^ transients during spontaneous action potentials.

The automatic optimization process provided a new set of parameters, which is reported in Table 1, fourth column. The simulated spontaneous APs and Ca^2+^ transients were in agreement with all the biomarker variability ranges (mean ± SD), as reported in the last column of Table 2. Figure 1 shows a comparison between the Paci2018 model (in solid blue) and the Paci2013 model (in dashed red). Figure 2 illustrates the simulated Ca^2+^ transients and traces from four illustrative cells: the comparison highlights that the simulated Ca^2+^ transient contour is fully in agreement with our experiments. In Figure 3 simulated spontaneous AP and Ca^2+^ traces in steady state are reported, together with the main ionic currents and concentrations. I_rel_ gating variables, together with the Ca^2+^ transients, are detailed in Figure 4. It shows that the Ca^2+^ release from SR is not directly dependent on the I_CaL_ activation, but on the cytosolic Ca^2+^ concentration Ca_i_, which rules the behavior of the RyR_o_, RyR_c_, and RyR_a_ gating variables.

### Current blocker simulations

**Figure 5:**
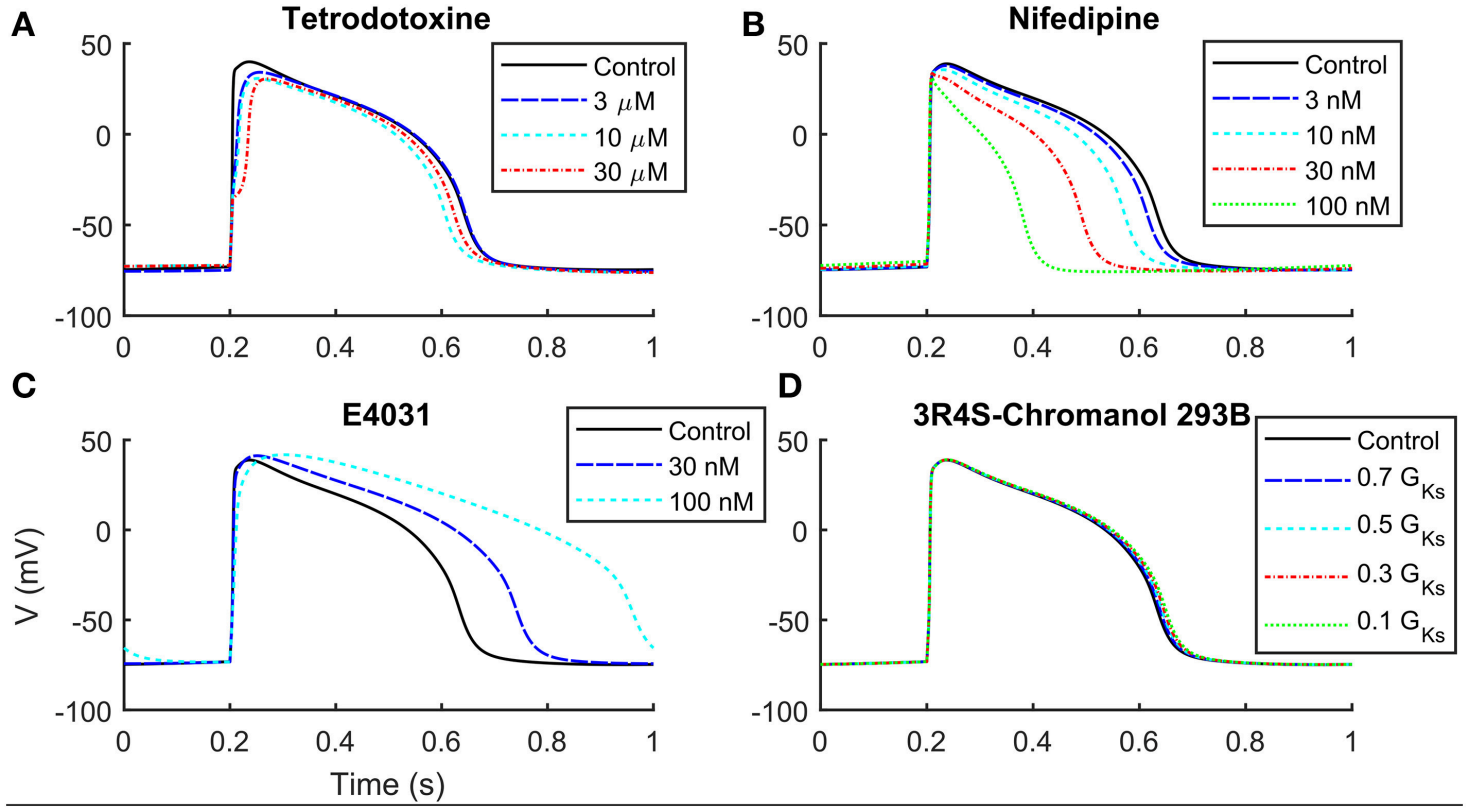
Simulation of current block effects on hiPSC-CMs paced at 1 Hz. **(A)** Tetrodotoxine blocks I_Na_, slowing down the upstroke phase. **(B)** Nifedipine blocks I_CaL_, shortening APD and triangulating AP profile. **(C)** E4031 blocks selectively I_Kr_, increasing APD. **(D)** I_Ks_ block by 3R4S-Chromanol 293B does not affect significantly the AP shape.

We compared in Table 3 the current blocker effects on the AP biomarkers (i) experimentally recorded by Ma et al. (2011), (ii) simulated by means of the Paci2013 model (Paci et al., 2013), and (iii) simulated by the Paci2018 model. Figure 5 shows that, despite the changes in the I_rel_ formulation and parameter optimization, the behavior of the Paci2018 model is still consistent with the Paci2013 simulations (Paci et al., 2013) and the experimental data by Ma et al. (2011). TTX affected the upstroke phase, reducing the AP V_max_ and delaying the AP Peak. NIFED, by acting on I_CaL_, reduced the Ca^2+^ influx through the cell membrane thus reducing the AP duration. Conversely, E4031 reduced the K^+^ efflux through the cell membrane, thus prolonging the AP. Finally, CHR showed little effect on the AP biomarkers, in agreement with the experiments.

**Table 3:** Quantitative effects of current blockers on stimulated APs.

**AP features**	**Experiments**		**Simulations**		
				**Paci2013**	**Paci2018**
V_max_	TTX 3 μM	41.2 ± 11.2%	18% G_Na_	26.2%	25.2%
	TTX 10 μM	16.7 ± 1.8%	6% G_Na_	23.5%	22.9%
	TTX 30 μM	16.8 ± 2.0%	2% G_Na_	20.7%	19.4%
APD_50_	E4031 30 nM	109.1 ± 3.7%	77% G_Kr_	124.8%	124.9%
	E4031 100 nM	113.4 ± 3.9%	50% G_Kr_	160.0%	175.1%
APD_90_	E4031 30 nM	140.3 ± 7.6%	77% G_Kr_	123.0%	123.4%
	E4031 100 nM	170.4 ± 13.6%	50% G_Kr_	151.4%	171.6%
APD_50_	Nifed 3 nM	84.6 ± 2.4%	93% G_CaL_	94.0%	95.3%
	Nifed 10 nM	70.3 ± 6.1%	79% G_CaL_	76.5%	85.2%
	Nifed 30 nM	65.7 ± 3.0%	56% G_CaL_	54.9%	65.3%
	Nifed 100 nM	45.4 ± 4.5%	28% G_CaL_	33.5%	37.7%
APD_90_	Nifed 3 nM	89.4 ± 1.0%	93% G_CaL_	96.5%	95.9%
	Nifed 10 nM	78.4 ± 4.4%	79% G_CaL_	84.3%	86.2%
	Nifed 30 nM	74.0 ± 2.3%	56% G_CaL_	68.8%	67.7%
	Nifed 100 nM	58.2 ± 5.4%	28% G_CaL_	51.4%	42.6%

*The experimental significant effects (p < 0.05) of current blockers on stimulated hiPSC-CMs from Ma et al. (2011) are compared with our previous model (Paci2013) and the current one (Paci2018). Experiments were performed on hiPSC-CMs paced at 1 Hz and are reported in terms of mean ± SE. Both experimental and simulated values are reported as percent of the control values. For direct comparison, the block levels simulated by the Paci2018 model were computed as in Paci et al. (2013) by means of dose-response curves: TTX (IC_50_ = 0.64 μM), Nifed (IC_50_ = 0.038 μM) and E4031 (IC_50_ = 100 nM)*.

### Delayed afterdepolarizations

**Figure 6:**
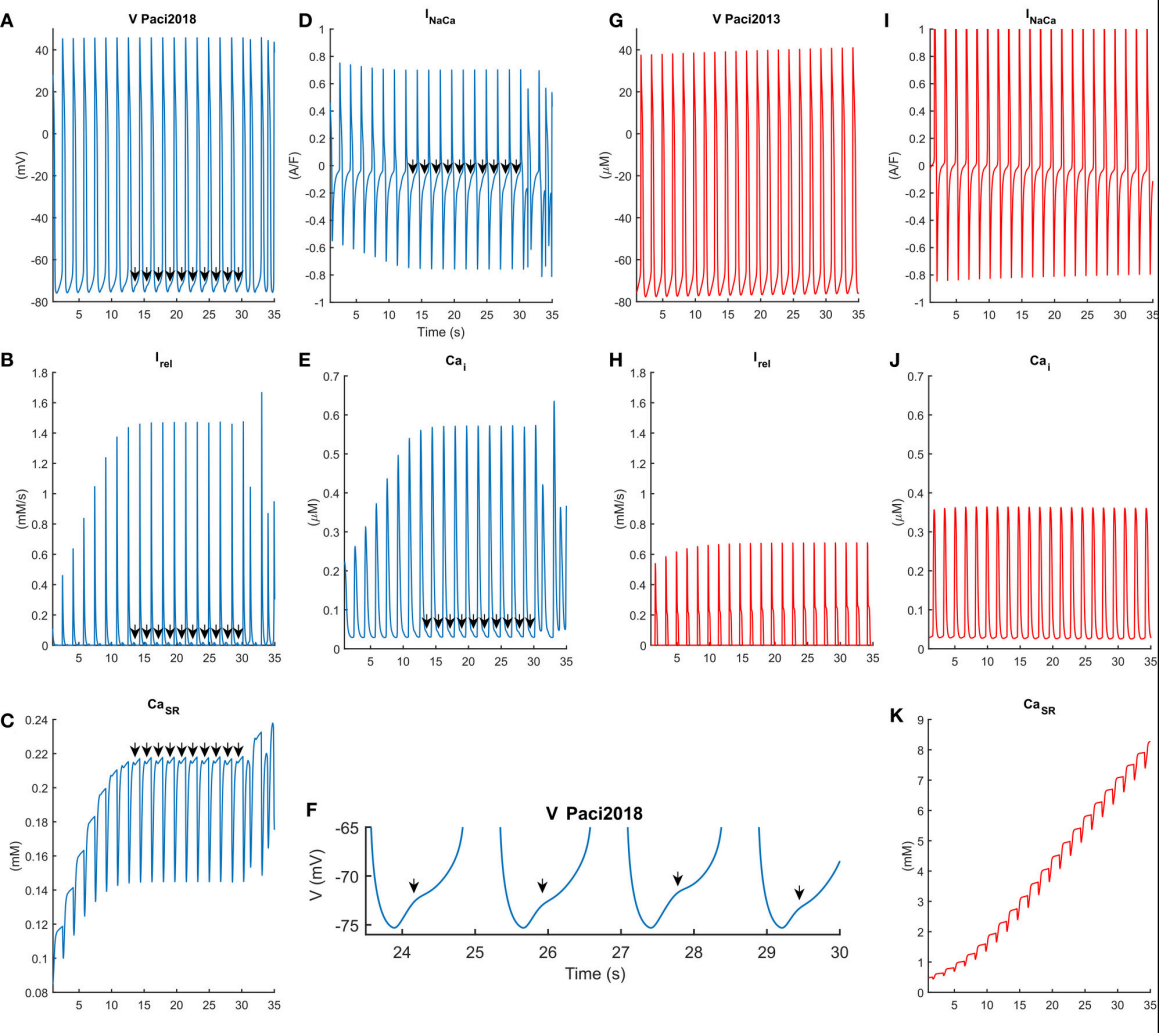
Comparison of the behavior of the Paci2018 (in blue, **A–F**) and the Paci2013 model (in red, **G–K**) in case of spontaneous APs and Ca^2+^ overload induced by hypercalcemia (Ca_o_ = 3.945 mM). In this condition both models can produce spontaneous APs, reported in **(A,G)**. The Paci2018 model shows a saturation of the sarcoplasmic Ca^2+^ concentration **(C)**, after which reactivation of I_rel_ **(B)** induces a distortion in the shape of the Ca^2+^ transients **(E)** and in the inward component of I_NaCa_ **(D)**, which corresponds to DADs in the membrane potential **(A,F)** in between *t* = [14, 30] s. At *t* = 30 s a strong spontaneous release of Ca^2+^ from SR is shown, which induces a spurious AP. On the contrary, the Paci2013 is not affected in these conditions and does not produce repolarization abnormalities **(G)**.

In Figure 6 we compared the behavior of the Paci2013 and the Paci2018 models in conditions of high extracellular Ca^2+^ concentration (Ca_o_ = 3.945 mM instead of 1.8 mM) and no external stimulation, i.e., both models produced spontaneous APs. As shown in Figure 6B, for *t* = [14, 30] s I_rel_ underwent a small reactivation, slowing down the decay of the Ca^2+^ transients (Figure 6E), affecting the inward component of I_NaCa_ (Figure 6D) and triggering DADs in the membrane potential (Figures 6A,F). Around *t* = 30 s, I_rel_ underwent a full reactivation, triggering an anticipated Ca^2+^ transient, which resulted in a full anticipated beat (Volders et al., 2000). We reported the extended AP and Ca^2+^ transient traces in the Supplementary Figure 1. Conversely, the old Paci2013 model did not show anomalies in the membrane potential (Figure 6G) or Ca^2+^ transients (Figure 6J) as consequence of the high extracellular Ca^2+^ concentration, while the Ca^2+^ in SR grew dramatically. The improvements introduced in the Paci2018 model allowed to simulate DADs not only in case of Ca^2+^ overload, but offered flexibility to simulate behaviors such as the spontaneous premature Ca^2+^ releases reported by Kim et al. (2015) in their Figures 2C, 4B and their Supplemental Figure S1B. We tested also the effect of a rate increment on the generation of DADs in case of hypercalcemia, by providing external pacing. In Supplementary Figure 2, we paced the Paci2018 model at its spontaneous basal rate (38 bpm, in solid blue) and at a basal rate increased by 50% (57 bpm, in dashed red). Despite the shorter diastolic depolarization phase, also at 57 bpm we obtained a fully developed DAD.

**Figure 7:**
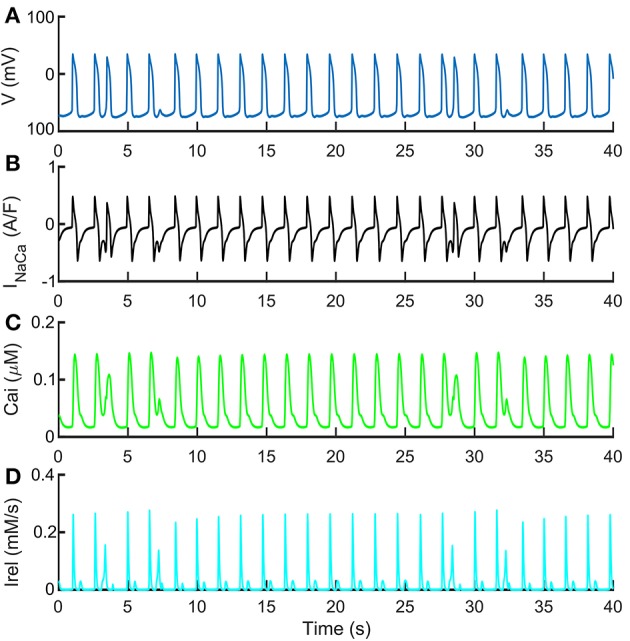
Repolarization abnormalities with standard extracellular Ca^2+^ concentrations. Such behavior was obtained with a control extracellular Ca^2+^ concentration Ca_o_ = 1.8 mM, shifting RyR_o,half_ and RyR_c,half_ by −0.002 and 0.002 mM respectively, doubling RyR_o_ time constant and reducing to half of its nominal value RyR_c_ time constant. These traces show the ability of the Paci2018 to simulate pathological conditions affecting the Ca^2+^ from SR. **(A)** Membrane potential. **(B)** I_NaCa_. **(C)** Cytosolic Ca^2+^ concentration. **(D)** I_rel_. A similar morphology of the cytosolic Ca^2+^ time-course is reported in Kim et al. (2015) in their Figure 4B.

Figure 7 shows the spontaneous APs, Ca^2+^ transients and I_NaCa_, together with the release Ca^2+^ flux (I_rel_), characterized with premature Ca^2+^ releases. These traces were obtained with a normal extracellular Ca^2+^ concentration (Ca_o_ = 1.8 mM) but simulating more “immature” RyR machinery (namely, by shifting RyR_o,half_ and RyR_c,half_ by −0.002 and 0.002 mM respectively, doubling RyR_o_ time constant and reducing to half of its nominal value RyR_c_ time constant). In these conditions of more “immature” RyR machinery, we tested also the effects of I_NaCa_ block on DADs generation. In Supplementary Figure 3, we tested three I_NaCa_ block levels (50, 70, and 90%) while pacing the Paci2018 model at 1 Hz. With no I_NaCa_ block (solid blue trace), APs show small DADs comparable to those reported in Figure 7 in *t* = [10, 25] s. By blocking 50% of I_NaCa_ (dashed orange trace), we observed that the amplitude of DADs increases. Finally, for the higher block levels, we did not observe any DAD.

### Comparison with other experimental data

In order to compare the Paci2018 model with the experimental data, we challenged our model in the following conditions: (i) I_f_ block by ivabradine, (ii) hyperkalemia, (iii) hypocalcemia.

**Figure 8:**
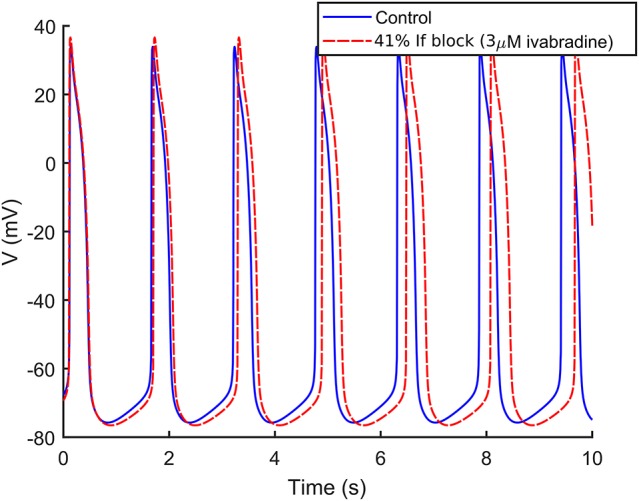
Effect of 3 μM ivabradine on the spontaneous APs. Administration of ivabradine (in dashed red) slightly slows down the rate of the spontaneous APs in comparison to control (in solid blue).

We simulated I_f_ block by 3 μM ivabradine as a 41% block of I_f_ maximum conductance (Yaniv et al., 2012; Koivumäki et al., 2018). As shown in Figure 8, I_f_ block induced only a slight reduction of the frequency of spontaneous APs (−2.3%), in agreement with (Kim et al., 2015), where 3 μM had virtually no effects on the spontaneous activity.

Kim et al. (2015) reported a slowdown of the spontaneous activity in conditions of hyperkalemia (from 4 to 8 mM the median frequency dropped from about 1.2 to 0.2 Hz) and a further increase in extracelluar K^+^ to 12 mM stopped the spontaneous activity. Our single cell model showed a similar trend (although with less sensitivity on K^+^): for K_o_ = 8 mM and K_o_ = 16 mM the Ca^2+^ transient spontaneous rate dropped by 11% and 15% respectively. An extracellular K^+^ concentration equal to 20 mM stopped the hiPSC-CM spontaneous activity as in Kim et al. (2015).

In the previous section, we tested the model in hypercalcemia conditions to simulate Ca^2+^ overload and the generation of DADs. We also challenged the model with an extracellular Ca^2+^ concentration of 0.1 mM (hypocalcemia) as in Kim et al. (2015): again we observed a trend qualitatively in agreement with the experimental data, since the rate of the spontaneous Ca^2+^ transients dropped by 17%.

## Discussion

In this paper we present an updated version of the Paci2013 model of ventricular-like hiPSC-CM (Paci et al., 2013), here named Paci2018, developed by exploiting new Ca^2+^ transients data and an automatic algorithm for the parameter optimization.

The Paci2018 model aims to overcome an important limitation of the Paci2013 model, i.e., the simple formulation of the Ca^2+^ release from SR, I_rel_, initially presented by the TenTusscher2004 model of human adult ventricular cardiomyocyte (ten Tusscher et al., 2004). The peculiarity of such I_rel_ is the direct link between the activation of I_CaL_ and the activation of I_rel_ (through the voltage-dependent activation gating variable d). This I_rel_ formulation was then chosen for its simplicity instead of more complex formulations, however it presumes I_rel_ to be directly dependent on the intracellular Ca^2+^ concentration and it prevents the model to be able to reproduce Ca^2+^-related anomalies such as DADs. As reported in Fink et al. (2011), models where the open probability of the RyR-sensitive channels is directly dependent on the opening of I_CaL_ are unable to produce DADs. Indeed, DADs start from a repolarized membrane, at potentials where the I_CaL_ gates are closed. Therefore, RyR-sensitive channels have to be able to open even when I_CaL_ is zero, which is not possible in the old I_rel_ formulation. Moreover, the presence of the c_rel_ constant in the TenTussher2004 I_rel_ formulation could allow a Ca^2+^ flux also from an empty SR, which is clearly not possible.

We chose a quite simple I_rel_ formulation, inspired by the Koivumäki2011 model of human adult atrial cardiomyocyte (Koivumäki et al., 2011), characterized by three gating variables. We did not choose more complex formulations, such as the Markov formulation in TenTusscher2006 model (ten Tusscher and Panfilov, 2006), since the latter was implemented mainly to correctly take into account the presence of T-tubules and the associated microdomains in the human adult ventricular cell. However, hiPSC-CMs produced so far with current differentiation protocols have not shown functional T-tubules (Li et al., 2013).

In order to keep the Paci2018 model the most consistent with its predecessor and with the Ma et al. dataset (Ma et al., 2011) of ionic currents and AP biomarkers, we did not change the formulation of I_Na_, I_CaL_, I_f_, I_to_, I_Kr_, I_Ks_, and I_K1_, which were carefully fitted for the development of the Paci2013 model. In order to tune the Paci2018 model on the new data of Ca^2+^ transients, we decided to act only on those parameters defining the formulation of currents for which the Ma et al. (2011) dataset did not provide direct experimental data.

The result is a new model where spontaneous APs match the Ma et al. dataset and where Ca^2+^ transients are in agreement with the new Ca^2+^ dataset. In this paper, we did not aim to propose only modifications to a previous model in order to simulate a specific phenomenon of interest, but we wanted to develop a new cardiac cell model to substantially increase modeling accuracy. Therefore, it was fundamental to check the Paci2018 model capability to reproduce all the experimental data used for the Paci2013 model validation. In detail, we tested the Paci2018 model's responses to the same prototypical current blockers used to validate the Paci2013 model, showing that the behavior of the two models is consistent and in agreement with the experimental data. We further validated the model to simulate the experimentally observed phenomenon of DADs and other specific results obtained in different experimental conditions, being able to replicate all these experiments. In particular, the Paci2018 model can reproduce Ca^2+^-related abnormalities such as DAD-like anomalies that were not available in old formalism. We observed that the small or full I_rel_ reactivations increase the cytosolic Ca^2+^ concentration and this produces an increased activity of the inward component of I_NaCa_, which then translates in DAD-like abnormalities in the membrane potential. This is particularly clear in Supplementary Figure 3, where for high levels of I_NaCa_ block (70 and 90%) we do not observe DADs. It is interesting to note that the 50% block is not enough to cancel DADs: in fact, the inward I_NaCa_ component is still strong enough and further enhanced by the cytosolic Ca^2+^ accumulation, due to the very same I_NaCa_ block (minimum diastolic Ca^2+^: 0.028 vs. 0.017 μM; average diastolic Ca^2+^: 0.060 vs. 0.027 μM, in case of 50% I_NaCa_ block or no I_NaCa_ block, respectively). This results in even larger DADs than in the no block case. Similar DAD-like abnormalities in the electrical properties of hiPSC-CMs were also observed in mutant hiPSC lines, such as those derived from patients with HCM (Ojala et al., 2016) or CPVT (Kujala et al., 2012). Of note, in this paper we do not aim to simulate these mutations, which would deserve a specific analysis by their own, but to provide a model which can enable there *in silico* modeling. Notably, the membrane potential and cytosolic Ca^2+^ time-courses obtained by our model are very similar to the experimental ones, as seen by comparison of our Figures 6, 7 with Figures 2C, 4B and S1 in Kim et al. (2015). In particular, Figure 4B in Kim et al. (2015) shows the effect of isoproterenol, a non-selective β adrenoreceptor agonist, which first induces small delayed spontaneous Ca^2+^ releases and then triggers repetitive high rate firing. In our Figures 6A,E we show a very similar pattern in conditions of hypercalcemia (see Supplementary Figure 1 for an expanded version of Ca^2+^ and AP time-courses), where the spontaneous Ca^2+^ releases, which trigger DADs, finally cause a transition to a higher rate of Ca^2+^ transients and APs.

To further validate our model, we challenged it to simulate specific conditions such as hyperkalemia and hypocalcemia, to compare its behavior to published experimental data. The model showed qualitative agreement with such experiments, in spite of the fact that the spontaneous rate reduction is stronger in the experiments. However, such differences can emerge from (i) the multicellular embryoid body culturing and measurements, i.e., on multicellular ensembles, and especially (ii) as consequence of the high inter-cell line and inter-laboratory variability showed by hiPSC-CMs (Knollmann, 2013), especially in beating rate and MDP. In fact, the embryoid bodies examined by Kim et al. (2015) exhibited negligible I_K1_ and a much depolarized MDP (−59.1 ± 3.3 mV), which facilitate the spontaneous electrical activity, as shown also by the high frequency of the spontaneous Ca^2+^ transients (1 Hz or higher) in control conditions. On the contrary, the experimental datasets we used in this paper showed slower spontaneous activity and more hyperpolarized MDP, as simulated by our model and reported in Table 2 (MDP = −75.8 mV, FREQ = 0.65 Hz).

We tested also the capability of the Paci2018 model to simulate early afterdepolarizations (EADs) in conditions of I_Kr_ block: the protocol and the results of the EAD simulations were reported in section 3 of the Supplementary Material. We observed that the Paci2018, as the old Paci2013 model, does not produce EADs. This is not surprising, since we designed the Paci2018 model carefully maintaining the same formulation for the ion currents that we fit on the experimental data by Ma et al. (2011), and that we used for the Paci2013 model (Paci et al., 2013). However, in our previous study (Paci et al., 2016), we demonstrated that I_Kr_ block triggered EADs in a population of *in silico* hiPSC-CM based on the Paci2013 model. Replicating here that study is out of the scope of this paper, however we decided to test if two parameter sets, which in Paci et al. (2016) triggered EADs, and one parameter set, which triggered repolarization failure, could induce repolarization abnormalities also in the Paci2018 model. Supplementary Figure 4 shows that, as expected, two derived models produced EADs and the third one repolarization failure. In particular, as reported in the Supplementary Table 1, these parameter sets showed (compared to baseline): (i) greater I_CaL_ (which corresponds to a greater Ca^2+^ influx during the repolarization phase in case of I_CaL_ reactivation); (ii) greater I_NaCa_ (which is inward during repolarization, thus promoting abnormalities in conditions of compromised repolarization as in case of I_Kr_ block), and (iii) smaller I_K1_ (corresponding to a smaller contribution to the membrane potential stabilization). It is also interesting to notice that the three parameter sets showed greater I_Kr_ compared to baseline: this suggests that in models where I_Kr_ is highly expressed, a 90% block can have more dramatic effects than in cells expressing smaller I_Kr_. This small test suggests, as we reported among the limitations, that the use of more refined modeling approaches, e.g., populations of *in silico* models, is advisable to simulate specific phenomena (in this case EADs as consequence of I_Kr_ block) in cell types characterized by high electrophysiological variability. In the perspective of offering researchers a tool to investigate the mechanisms of ventricular arrhythmia development in hiPSC-CMs, we ran also two preliminary tests about the occurrence of alternans or alternans-like patterns in the Paci2018 model (Methods and Results are detailed in section 4 of the Supplementary Material). When pacing the Paci2018 model at 120 bpm (2 Hz), the membrane potential exhibited an alternans-like pattern, characterized by a full AP followed by a smaller one (Supplementary Figure 5A). We tested also the effect of very high pacing rate (200 bpm, 3.33 Hz) in ischemia-like conditions (Supplementary Figure 5B): in this case, the membrane potential was strongly depolarized (−60 mV) and exhibited 2:1 alternans. We consider these results valuable also for more advanced studies regarding arrhythmias propagation in case of extension of the simulations to a monodimensional strand or a bidimensional patch.

Overall, we believe that the here presented Paci2018 model substantially increases hiPSC-CM modeling accuracy. Of course, as every *in silico* model, also the Paci2018 is an approximated description of a real system: consequently, new mechanisms can be explored and improved. This is especially true for such a complex cellular system as hiPSC-CM, which is not fully characterized yet. The main limitation that affects the Paci2018 model, as well the other single cell *in silico* models available in literature, is the fact that the simulated behavior belongs to a theoretical cell, built on averaged experimental data from many cells. In spite of our efforts to build the Paci2018 in a rigorous way, we do not have the presumption to consider it representative of all the hiPSC-CMs available nowadays, both from commercial lines and laboratory-specific lines. This is particularly true in case of an *in vitro* model like hiPSC-CMs, since the same experiments that we need to build an *in silico* model are extremely variable, as reported by multiple studies (Knollmann, 2013; Lu et al., 2015; Paci et al., 2017). As an additional example, in Lu et al. (2015) the authors succeeded in culturing iCell hiPSC-CMs that showed limited biomarker variability. Nevertheless, their cells showed way slower spontaneous beating rate than the cells we used to build our model (~15 bpm vs. ~38 bpm), despite both datasets were recorded at the same 37°C temperature. In order to have a better translation from *in silico* to *in vitro* models and finally to (pre)clinical applications, more refined modeling techniques exist, e.g., the population of *in silico* models (Britton et al., 2013; Passini et al., 2017). In addition to this, in terms of translation of drug test results from hiPSC-CMs to clinical applications, more challenges emerged recently. For example, in Abi-Gerges et al. (2017), testing a panel of 30 drugs on non-paced hiPSC-CMs provided on one hand good predictivity of pro-arrhythmic events, but on the other hand limited predictivity as an early QT screening. Therefore, in spite the high potential of hiPSC-CMs as *in vitro* models for diseases and drug tests, direct translation of results is still a work in progress, as indicated by the fact that advances in phenotype selection, cell maturation and combined recording platforms are currently being made. One inherent limitation of the Paci2018 model consists in the use of two different datasets of experimental data, one for the AP and ionic current data and the other for Ca^2+^ data. This is clear, e.g., by comparing the mean values of APD_90_ and Ca^2+^ transient DURATION biomarkers: 414.7 and 804.5 ms, respectively. It is known that high affinity Ca^2+^ indicators, such as Fluo-4, can artificially prolong the Ca^2+^ transients and that Ca^2+^ transients last more than APs (Lee et al., 2012); however, the aforementioned Ca^2+^ transient DURATION value is substantially longer than APD_90_. Therefore, we tried to find an acceptable tradeoff by means of the parameter optimization algorithm, in order to fulfill all the experimental ranges of both the AP and Ca^2+^ transient biomarkers. In particular, in the cost function we chose heavier weights (w_i_ = 2) only for two critical biomarkers which characterize hiPSC-CMs, i.e., MDP (depolarized compared to adult cardiomyocytes) and the rate of the spontaneous APs. For all the other biomarkers, the weights were set to one. The tradeoff obtained by the parameter optimization produced a model that simulates Ca^2+^ transients whose DURATION is 617.8 ms. Despite this value lies within the experimental range 804.5 ± 188.0 ms, it is very close to its lower bound. Conversely, the DT_9010_ biomarker properly lies within its experimental range, although slightly smaller than the experimental mean DT_9010_ (409.8 ± 100.1 vs. 367.6 ms). The inset included in Figure 2 shows only the decay part (from 90 to 10% of the transient amplitude) of the experimental and simulated transients reported in the main Figure 2: our simulated decay lies within the illustrative experimental traces, as well as DT_9010_ of the simulated transient is included in the experimental interval. Of note, the experimental traces reported in Figure 2 are purely illustrative of our experiments: the global quantitative comparison between experiments and simulations is done in Table 2 in terms of biomarkers. It is also known that temperature affects the spontaneous electrical activity of hiPSC-CMs, e.g., in Laurila et al. (2016) the authors showed how the temperature increments and reductions accelerate or slow down hiPSC-CM spontaneous beating, respectively. The fact that the AP and Ca^2+^ transient experimental datasets, chosen to develop the Paci2018 model, were recorded at the same temperature (35–37°C) further mitigates the differences between the two datasets. Another potential limitation of our model is related to its simple structure, consisting of two compartments only (cytosol and SR). Such compartmentalization does not allow to simulate a spatial inhomogeneous Ca^2+^ distribution, as conversely recently done by others (Koivumäki et al., 2018). We preferred to keep the model formulation simple, without entering in such detailed description, since it would have required a more complex mathematical formulation, a more detailed knowledge of the subcellular organization of hiPSC-CMs and, consequently, a more challenging parameter identification and higher simulation time. However, our formulation is successful in simulating the most important electrophysiological mechanisms at the whole cell level, including DADs elicited from different kind of stresses, without resorting to random RyR openings, as done in Koivumäki et al. (2018). A final limitation of the Paci2018 model is that it describes only the electrophysiology and the Ca^2+^ handling, but it does not take into consideration contractility. In literature, a few models of myofilament are available (Negroni and Lascano, 2008; Rice et al., 2008; Negroni et al., 2015). For instance, in Negroni et al. (2015) the authors presented an integrated rabbit ventricular cardiomyocyte model, which included also the mathematical description of force generation and the myofilament Ca^2+^ kinetics. However, the sarcomeric structure of hiPSC-CMs is immature and the myofibrils in hiPSC-CMs are oriented in multiple directions within the cell: in Bedada et al. (2016) a higher level of sarcomeric organization was reached only by repopulating hiPSC-CMs into a biological cardiac matrix. We acknowledge the potential of an integration of the myofilament description in the Paci2018, nevertheless the translation from a different species model of, e.g., rabbit cardiomyoycte to a hiPSC-CM model is not elementary and out of the scope of this paper. Future developments of this work will include using the Paci2018 model to ran *in silico* drug tests and to ran monodimensional and bidimensional simulations, as done, e.g., by Raphel et al. with the Paci2013 model (Raphel et al., 2017).

In conclusion, in this work we present an updated and more versatile version of our hiPSC-CM *in silico* model, based on a new dataset of electrophysiological data. Our model can represents the basis for new *in silico* studies on the effects of drugs or mutations (e.g., CPVT), which affect the Ca^2+^ handling in hiPSC-CMs. Due to its relatively light formulation (23 ordinary differential equations), our model is suitable also for very large studies on *in silico* populations, e.g., to support screening of different drug/compounds at various concentrations.

## Author contributions

All the authors conceived and designed the study. R-PP and KP performed the *in vitro* measurements and analyzed the *in vitro* data. MP and DC performed the *in silico* model development and validation. MP and SS analyzed the *in silico* data, prepared the figures and drafted the manuscript. All the authors interpreted the results and revised the manuscript.

### Conflict of interest statement

The authors declare that the research was conducted in the absence of any commercial or financial relationships that could be construed as a potential conflict of interest. The reviewer JB and handling Editor declared their shared affiliation.
