# Supplementary material for: Automatic Optimization of an in Silico Model of Human iPSC Derived Cardiomyocytes Recapitulating Calcium Handling Abnormalities
Source: Front Physiol. 2018 Jun 26;9:709. doi: 10.3389/fphys.2018.00709 (PMC6028769; doi:10.3389/fphys.2018.00709)
Supplement: Supplementary file 1 [file Data_Sheet_1.pdf]

Supplementary Material

Automatic optimization of an *in silico* model of human iPSC derived cardiomyocytes recapitulating calcium handling abnormalities

Michelangelo Paci*, Risto-Pekka Pölönen, Dario Cori, Kirsi Penttinen, Katriina Aalto-Setälä, Stefano Severi, Jari Hyttinen

*** Correspondence:** Michelangelo Paci: michelangelo.paci@tut.fi

1 Formulation of the hiPSC-CM model

V: membrane potential in *V*. *VmV*: membrane potential in *mV*. *time*: simulation time in *s*.

All the time constants in *ms*. All the ionic concentrations in *mM*. All the reversal potentials in *V*.

1.1 Extracellular ionic concentrations

$Na_o = 151\ (mM)$

$K_o = 5.4\ (mM)$

$Ca_o = 1.8\ (mM)$

1.2 Cell size and dimensions

$C_m = 98.7109e-12\ (F)$

$V_c = 8800e-18\ (m^3)$

$V_{sr} = 583.73e-18\ (m^3)$

1.3 Maximum conductances and currents

$g_{Na} = 3671.2302\ (S/F)$

$g_{NaL} = 17.25\ (S/F)$

$g_{CaL} = 8.635702e-5(m^3/(F \times s))$

$g_{to} = 29.9038\,(S/F)$

$g_{Kr} = 29.8667(S/F)$

$g_{Ks} = 2.041\,(S/F)$

$g_{K1} = 28.1492\,(S/F)$

$g_{f} = 30.10312\,(S/F)$

$P_{NaK} = 2.6351\,(A/F)$

$K_{NaCa} = 3917.0463\,(A/F)$

$I_{rel,\max} = 62.5434\,(mM/s)$

$V_{\max,up} = 0.5113\,(mM/s)$

$I_{leak,\max} = 4.7279\text{e-}4\,(1/s)$

$g_{pCa} = 0.4125(A/F)$

$g_{bNa} = 0.95(S/F)$

$g_{bCa} = 0.727272(S/F)$

1.4 Other constants

$Buf_{c} = 0.25\,(mM)$

$Buf_{sr} = 10\,(mM)$

$K_{buf_{c}} = 0.001(mM)$

$K_{buf_{sr}} = 0.3\,(mM)$

$K_{up} = 3.1928\text{e-}4\,(mM)$

$K_{pCa} = 0.0005\,(mM)$

$F = 96485.3415\,(C/mol)$

$R = 8.314472\ (J/(mol \times K))$

$T = 310\ (K)$

$L_0 = 0.025\ (dimensionless)$

$Q = 2.3\ (dimensionless)$

$P_{kna} = 0.03\ (dimensionless)$

$K_{sat} = 0.1\ (dimensionless)$

$K_{mCa} = 1.38\ (mM)$

$K_{mNai} = 87.5\ (mM)$

$\alpha = 2.5371\ (dimensionless)$

$\gamma = 0.35\ (dimensionless)$

$K_{mNa} = 40\ (mM)$

$K_{mK} = 1\ (mM)$

$RyR_{a1} = 0.05354\ (\mu M)$

$RyR_{a2} = 0.0488\ (\mu M)$

$RyR_{a,half} = 0.02427\ (\mu M)$

$RyR_{o,half} = 0.01042\ (\mu M)$

$RyR_{c,half} = 0.00144\ (\mu M)$

1.5 Initial conditions of state variables

$h_0 = 0.75\ (dimensionless)$

$j_0 = 0.75\ (dimensionless)$

$m_0 = 0\ (dimensionless)$

$mL_0 = 0\ (dimensionless)$

$hL_0 = 0.75 \ (dimensionless)$

$d_0 = 0 \ (dimensionless)$

$fCa_0 = 1 \ (dimensionless)$

$f1_0 = 1 \ (dimensionless)$

$f2_0 = 1 \ (dimensionless)$

$r_0 = 0 \ (dimensionless)$

$q_0 = 1 \ (dimensionless)$

$Xr1_0 = 0 (dimensionless)$

$Xr2_0 = 1 \ (dimensionless)$

$Xs_0 = 0 (dimensionless)$

$Xf_0 = 0.1 (dimensionless)$

$RyRa_0 = 0.3 \ (dimensionless)$

$RyRo_0 = 0.9 \ (dimensionless)$

$RyRc_0 = 0.1 \ (dimensionless)$

$V_0 = -0.070 (V)$

$Na_i = 9.2 (mM)$

$K_i = 150 \ (mM)$

$Ca_i = 0.0002 \ (mM)$

$Ca_{SR} = 0.32 \ (mM)$

1.6 Membrane Potential

$$\frac{\mathrm{d}V}{\mathrm{d}time} = -I_{ion} = -\left(I_{K1} + I_{to} + I_{Kr} + I_{Ks} + I_{CaL} + I_{NaK} + I_{Na} + I_{NaL} + I_{NaCa} + I_{p_{Ca}} + I_f + I_{bNa} + I_{bCa}\right) \quad \text{(S1)}$$

1.7 Na^+ current, I_{Na}

$$I_{Na} = g_{Na} \cdot m^3 \cdot h \cdot j \cdot (V - E_{Na}) \tag{S2}$$

1.7.1 I_{Na}, *h* gate

$$h_{inf} = \frac{1}{\sqrt{\left(1 + e^{\frac{VmV + 72.1}{5.7}}\right)}} \tag{S3}$$

$$\alpha_h = 0.057 \cdot e^{\frac{-VmV - 80}{6.8}} \tag{S4}$$

$$\beta_h = 2.7 \cdot e^{0.079 \cdot VmV} + 3.1 \times 10^5 \cdot e^{0.3485 \cdot VmV} \tag{S5}$$

$$\tau_h = \begin{cases} \dfrac{1.5}{\alpha_h + \beta_h}, & if\ VmV < -38.5 (mV) \\ 2.54205, & if\ VmV \geq -38.5 (mV) \end{cases} \tag{S6}$$

$$\frac{\mathrm{d}h}{\mathrm{d}time} = 1000 \cdot \frac{h_{inf} - h}{\tau_h} \tag{S7}$$

1.7.2 I_{Na}, *j* gate

$$j_{inf} = h_{inf} \tag{S8}$$

$$\alpha_j = \begin{cases} \dfrac{\left(-25428 \cdot e^{0.2444 \cdot VmV} - 6.948e-6 \cdot e^{-0.04391 \cdot VmV}\right) \cdot (VmV + 37.78)}{1 + e^{0.311 \cdot (VmV + 79.23)}}, & if\ VmV < -40\ (mV) \\ 0, & otherwise \end{cases} \tag{S9}$$

$$\beta_j = \begin{cases} \dfrac{0.02424 \cdot e^{-0.01052 \cdot VmV}}{1 + e^{-0.1378 \cdot (VmV + 40.14)}}, & if\ VmV < -40\ (mV) \\ \dfrac{0.6 \cdot e^{0.057 \cdot VmV}}{1 + e^{-0.1 \cdot (VmV + 32)}}, & otherwise \end{cases} \tag{S10}$$

$$\tau_j = \frac{7}{\alpha_j + \beta_j} \tag{S11}$$

$$\frac{\mathrm{d}j}{\mathrm{d}time} = 1000 \cdot \frac{j_{inf} - j}{\tau_j} \tag{S12}$$

1.7.3 I_{Na}, *m* gate

$$m_{inf} = \frac{1}{\left(1 + e^{\frac{-34.1 - VmV}{5.9}}\right)^{1/3}} \tag{S13}$$

$$\alpha_m = \frac{1}{1 + e^{\frac{-60 - VmV}{5}}} \tag{S14}$$

$$\beta_m = \frac{0.1}{1 + e^{\frac{VmV + 35}{5}}} + \frac{0.1}{1 + e^{\frac{VmV - 50}{200}}} \tag{S15}$$

$$\tau_m = \alpha_m \cdot \beta_m \tag{S16}$$

$$\frac{\mathrm{d}m}{\mathrm{d}time} = 1000 \cdot \frac{m_{inf} - m}{\tau_m} \tag{S17}$$

1.8 Late Na^+ current, I_{NaL}

$$I_{Na} = g_{NaL} \cdot mL^3 \cdot hL \cdot \left(V - E_{Na}\right) \tag{S18}$$

1.8.1 I_{NaL}, *hL* gate

$$hL_{inf} = \frac{1}{\left(1 + e^{\frac{VmV + 87.61}{7.488}}\right)} \tag{S19}$$

$$\tau_{hL} = 200 \ \ (ms) \tag{S20}$$

$$\frac{\mathrm{d}hL}{\mathrm{d}time} = 1000 \cdot \frac{hL_{inf} - hL}{\tau_{hL}} \tag{S21}$$

1.8.2 I_{NaL}, *mL* gate

$$mL_{inf} = \frac{1}{\left(1 + e^{\frac{-VmV - 42.85}{5.264}}\right)} \tag{S22}$$

$$\alpha_{mL} = \frac{1}{1 + e^{\frac{-60 - VmV}{5}}} \tag{S23}$$

$$\beta_{mL} = \frac{0.1}{1+e^{\frac{(VmV+35)}{5}}} + \frac{0.1}{1+e^{\frac{(VmV-50)}{200}}} \tag{S24}$$

$$\tau_{mL} = \alpha_{mL} \cdot \beta_{mL} \tag{S25}$$

$$\frac{\mathrm{d}mL}{\mathrm{d}time} = 1000 \cdot \frac{mL_{inf} - mL}{\tau_{mL}} \tag{S26}$$

1.9 L-type Ca^{2+} current, I_{CaL}

$$I_{CaL} = \frac{g_{CaL} \cdot 4 \cdot V \cdot F^2}{R \cdot T} \cdot \frac{\left(Ca_i \cdot e^{\frac{2 \cdot V \cdot F}{R \cdot T}} - 0.341 \cdot Ca_o \right)}{e^{\frac{2 \cdot V \cdot F}{R \cdot T}} - 1} \cdot d \cdot f1 \cdot f2 \cdot fCa \tag{S27}$$

1.9.1 I_{CaL}, *d* gate

$$d_{inf} = \frac{1}{1+e^{\frac{-VmV-9.1}{7}}} \tag{S28}$$

$$\alpha_d = \frac{1.4}{1+e^{\frac{-35-VmV}{13}}} + 0.25 \tag{S29}$$

$$\beta_d = \frac{1.4}{1+e^{\frac{VmV+5}{5}}} \tag{S30}$$

$$\gamma_d = \frac{1}{1+e^{\frac{50-VmV}{20}}} \tag{S31}$$

$$\tau_d = \alpha_d \cdot \beta_d + \gamma_d \tag{S32}$$

$$\frac{\mathrm{d}d}{\mathrm{d}time} = 1000 \cdot \frac{d_{inf} - d}{\tau_d} \tag{S33}$$

1.9.2 I_{CaL}, f_{Ca} gate

$$\alpha_{fCa} = \frac{1}{1+\left(\frac{Ca_i}{0.0006}\right)^8} \tag{S34}$$

$$\beta_{fCa} = \frac{0.1}{1 + e^{\frac{Ca_i - 0.0009}{0.0001}}} \quad \text{(S35)}$$

$$\gamma_{fCa} = \frac{0.3}{1 + e^{\frac{Ca_i - 0.00075}{0.0008}}} \quad \text{(S36)}$$

$$fCa_{inf} = \frac{\alpha_{fCa} + \beta_{fCa} + \gamma_{fCa}}{1.3156} \quad \text{(S37)}$$

$$\tau_{fCa} = 2\ (ms) \quad \text{(S38)}$$

$$\frac{\mathrm{d}fCa}{\mathrm{d}t} = constfCa \cdot 1000 \cdot \frac{fCa_{inf} - fCa}{\tau_{fCa}} \quad \text{(S39)}$$

$$constfCa = \begin{cases} 0, & if\ fCa_{inf} > fCa\ and\ VmV > -60\ (mV) \\ 1, & otherwise \end{cases} \quad \text{(S40)}$$

1.9.3 I_{CaL}, *f1* gate

$$f1_{inf} = \frac{1}{1 + e^{\frac{VmV + 26}{3}}} \quad \text{(S41)}$$

$$\tau_{f1} = \left(1102.5 \cdot e^{-\left[\frac{(VmV+27)^2}{15}\right]^2} + \frac{200}{1 + e^{\frac{13 - VmV}{10}}} + \frac{180}{1 + e^{\frac{VmV + 30}{10}}} + 20 \right) \cdot \begin{cases} \left(1 + 1433 \cdot (Ca_i - 50e - 6)\right), & if\ \frac{df1}{dtime} > 0 \\ 1, & otherwise \end{cases}$$

(S42)

$$\frac{\mathrm{d}f1}{\mathrm{d}time} = 1000 \cdot \frac{f1_{inf} - f1}{\tau_{f1}} \quad \text{(S43)}$$

1.9.4 I_{CaL}, *f2* gate

$$f2_{inf} = \frac{0.67}{1 + e^{\frac{VmV + 32}{4}}} + 0.33 \quad \text{(S44)}$$

$$\tau_{f2} = 600 \cdot e^{-\frac{(VmV+25)^2}{170}} + \frac{31}{1 + e^{\frac{25 - VmV}{10}}} + \frac{16}{1 + e^{\frac{VmV + 30}{10}}} \quad \text{(S45)}$$

$$\frac{\mathrm{d}f2}{\mathrm{d}time} = 1000 \cdot \frac{f2_{inf} - f2}{\tau_{f2}} \tag{S46}$$

1.10 Transient outward current, I_{to}

$$I_{to} = g_{to} \cdot r \cdot q \cdot (V - E_K) \tag{S47}$$

1.10.1 I_{to}, *r* gate

$$r_{inf} = \frac{1}{1 + e^{\frac{22.3 - VmV}{18.75}}} \tag{S48}$$

$$\tau_r = \frac{14.40516}{1.037e^{0.09 \cdot (VmV + 30.61)} + 0.369e^{-0.12 \cdot (VmV + 23.84)}} + 2.75352 \tag{S49}$$

$$\frac{\mathrm{d}r}{\mathrm{d}time} = 1000 \cdot \frac{r_{inf} - r}{\tau_r} \tag{S50}$$

1.10.2 I_{to}, *q* gate

$$q_{inf} = \frac{1}{1 + e^{\frac{VmV + 53}{13}}} \tag{S51}$$

$$\tau_q = \frac{39.102}{0.57e^{-0.08 \cdot (VmV + 44)} + 0.065e^{0.1 \cdot (VmV + 45.93)}} + 6.06 \tag{S52}$$

$$\frac{\mathrm{d}q}{\mathrm{d}time} = 1000 \cdot \frac{q_{inf} - q}{\tau_q} \tag{S53}$$

1.11 Rapid delayed rectifier K^+ current, I_{Kr}

$$I_{Kr} = g_{Kr} \cdot \sqrt{\frac{K_o}{5.4}} \cdot Xr1 \cdot Xr2 \cdot (V - E_K) \tag{S54}$$

1.11.1 I_{Kr}, *Xr1* gate

$$V_{half,Xr1} = 1000 \cdot \left(-\frac{R \cdot T}{F \cdot Q} \cdot ln \left(\frac{\left(1 + \frac{Ca_o}{2.6}\right)^4}{L_0 \cdot \left(1 + \frac{Ca_o}{0.58}\right)^4} \right) - 0.019 \right) \tag{S55}$$

$$Xr1_{inf} = \frac{1}{1+e^{\frac{V_{half,Xr1}-VmV}{4.9}}} \quad \text{(S56)}$$

$$\alpha_{Xr1} = \frac{450}{1+e^{\frac{-45-VmV}{10}}} \quad \text{(S57)}$$

$$\beta_{Xr1} = \frac{6}{1+e^{\frac{VmV+30}{11.5}}} \quad \text{(S58)}$$

$$\tau_{Xr1} = \alpha_{Xr1} \cdot \beta_{Xr1} \quad \text{(S59)}$$

$$\frac{\mathrm{d}Xr1}{\mathrm{d}time} = 1000 \cdot \frac{Xr1_{inf} - Xr1}{\tau_{Xr1}} \quad \text{(S60)}$$

1.11.2 I_{Kr}, *Xr2* gate

$$Xr2_{inf} = \frac{1}{1+e^{\frac{VmV+88}{50}}} \quad \text{(S61)}$$

$$\alpha_{Xr2} = \frac{3}{1+e^{\frac{-60-VmV}{20}}} \quad \text{(S62)}$$

$$\beta_{Xr2} = \frac{1.12}{1+e^{\frac{VmV-60}{20}}} \quad \text{(S63)}$$

$$\tau_{Xr2} = \alpha_{Xr2} \cdot \beta_{Xr2} \quad \text{(S64)}$$

$$\frac{\mathrm{d}Xr2}{\mathrm{d}time} = 1000 \cdot \frac{Xr2_{inf} - Xr2}{\tau_{Xr2}} \quad \text{(S65)}$$

1.12 Slow delayed rectifier K^+ current, I_{Ks}

$$I_{Ks} = g_{Ks} \cdot Xs^2 \cdot \left(1 + \frac{0.6}{1+\left(\frac{3.8e-5}{Ca_i}\right)^{1.4}} \right) \cdot \left(V - E_{Ks}\right) \quad \text{(S66)}$$

1.12.1 I_{Ks}, *Xs* gate

$$Xs_{inf} = \frac{1}{1+e^{\frac{-20-VmV}{16}}} \tag{S67}$$

$$\alpha_{Xs} = \frac{1100}{\sqrt{1+e^{\frac{-10-VmV}{6}}}} \tag{S68}$$

$$\beta_{Xs} = \frac{1}{1+e^{\frac{VmV-60}{20}}} \tag{S69}$$

$$\tau_{Xs} = \alpha_{Xs} \cdot \beta_{Xs} \tag{S70}$$

$$\frac{\mathrm{d}Xs}{\mathrm{d}time} = 1000 \cdot \frac{Xs_{inf} - Xs}{\tau_{Xs}} \tag{S71}$$

1.13 Inward rectifier K^+ current, I_{K1}

$$\alpha_{K1} = \frac{3.91}{1+e^{0.5942\cdot(VmV-1000\cdot E_K-200)}} \tag{S72}$$

$$\beta_{K1} = \frac{-1.509 \cdot e^{0.0002\cdot(VmV-1000\cdot E_K+100)} + e^{0.5886\cdot(VmV-1000\cdot E_K-10)}}{1+e^{0.4547\cdot(VmV-1000\cdot E_K)}} \tag{S73}$$

$$XK1_{inf} = \frac{\alpha_{K1}}{\alpha_{K1} + \beta_{K1}} \tag{S74}$$

$$I_{K1} = g_{K1} \cdot XK1_{inf} \cdot \sqrt{\frac{K_o}{5.4}} \cdot \left(V - E_K\right) \tag{S75}$$

1.14 Hyperpolarization activated funny current, I_f

$$I_f = g_f \cdot Xf \cdot \left(V - E_f\right) \tag{S76}$$

1.14.1 I_f, *Xf* gate

$$Xf_{inf} = \frac{1}{1+e^{\frac{VmV+77.85}{5}}} \tag{S77}$$

$$\tau_{Xf} = \frac{1900}{1+e^{\frac{VmV+15}{10}}} \tag{S78}$$

$$\frac{dXf}{dtime} = 1000 \cdot \frac{Xf_{inf} - Xf}{\tau_{Xf}} \tag{S79}$$

1.14.2 Na^+ component of I_f

$$I_{fNa} = 0.42 \cdot g_f \cdot Xf \cdot \left(V - E_{Na}\right) \tag{S80}$$

1.15 Na^+/K^+ pump current, I_{NaK}

$$I_{NaK} = P_{NaK} \frac{\frac{K_o}{K_o + K_{mK}} \cdot \frac{Na_i}{Na_i + K_{mNa}}}{1 + 0.1245 \cdot e^{\frac{-0.1 \cdot V \cdot F}{R \cdot T}} + 0.0353 \cdot e^{\frac{-V \cdot F}{R \cdot T}}} \tag{S81}$$

1.16 Na^+/Ca^{2+} exchanger current, I_{NaCa}

$$I_{NaCa} = \frac{K_{NaCa} \cdot \left(e^{\frac{\gamma \cdot V \cdot F}{R \cdot T}} \cdot Na_i^{\,3} \cdot Ca_o - e^{\frac{(\gamma - 1) \cdot V \cdot F}{R \cdot T}} \cdot Na_o^{\,3} \cdot Ca_i \cdot \alpha \right)}{\left(K_{mNai}^{\,3} + Na_o^{\,3}\right) \cdot \left(K_{mCa} + Ca_o\right) \cdot \left(1 + K_{sat} \cdot e^{\frac{(\gamma - 1) \cdot V \cdot F}{R \cdot T}}\right)} \tag{S82}$$

1.17 Ca^{2+} dynamics

$$RyR_{CaSR} = 1 - \frac{1}{1+e^{\frac{Ca_{SR}-0.3}{0.1}}} \tag{S83}$$

$$RyRa_{\inf} = RyR_{a1} - \frac{RyR_{a2}}{1+e^{\frac{1000 \cdot Ca_i - RyR_{a,half}}{0.0082}}} \tag{S84}$$

$$\tau_{RyRa} = 1000\,(ms) \tag{S85}$$

$$\frac{dRyRa}{dtime} = 1000 \cdot \frac{RyRa_{\inf} - RyRa}{\tau_{RyRa}} \tag{S86}$$

$$RyRo_{\inf} = 1 - \frac{1}{1 + e^{\frac{1000 \cdot Ca_i - (RyRa + RyR_{o,half})}{0.003}}} \quad \text{(S87)}$$

$$\tau_{RyRo} = \begin{cases} 18.75, & if \left(RyRo_{inf} \geq RyRo \right) \\ 1.875, & otherwise \end{cases} \quad \text{(S88)}$$

$$\frac{dRyRo}{dtime} = 1000 \cdot \frac{RyRo_{\inf} - RyRo}{\tau_{RyRo}} \quad \text{(S89)}$$

$$RyRc_{\inf} = \frac{1}{1 + e^{\frac{1000 \cdot Ca_i - (RyRa + RyR_{c,half})}{0.001}}} \quad \text{(S90)}$$

$$\tau_{RyRc} = \begin{cases} 175.0, & if \left(RyRc_{inf} \geq RyRc \right) \\ 87.5, & otherwise \end{cases} \quad \text{(S91)}$$

$$\frac{dRyRc}{dtime} = 1000 \cdot \frac{RyRc_{\inf} - RyRc}{\tau_{RyRc}} \quad \text{(S92)}$$

$$I_{rel} = I_{rel,\max} \cdot RyR_{CaSR} \cdot RyRo \cdot RyRc \cdot (Ca_{SR} - Ca_i) \quad \text{(S93)}$$

$$I_{up} = \frac{V_{\max,up}}{1 + \frac{K_{up}^2}{Ca_i^2}} \quad \text{(S94)}$$

$$I_{leak} = I_{leak,\max} \cdot \left(Ca_{SR} - Ca_i \right) \quad \text{(S95)}$$

$$Ca_{i_{bufc}} = \frac{1}{1 + \frac{Buf_c \cdot K_{buf_c}}{(Ca_i + K_{buf_c})^2}} \quad \text{(S96)}$$

$$Ca_{sr_{bufsr}} = \frac{1}{1 + \frac{Buf_{sr} \cdot K_{buf_{sr}}}{(Ca_{SR} + K_{buf_{sr}})^2}} \quad \text{(S97)}$$

$$\frac{\mathrm{d}Ca_i}{\mathrm{d}time} = Ca_{i_{bufc}} \cdot \left(I_{leak} - I_{up} + I_{rel} - \frac{\left(I_{CaL} + I_{bCa} + I_{p_{Ca}} - 2 \cdot I_{NaCa} \right)}{2 \cdot V_c \cdot F} \cdot C_m \right) \quad \text{(S98)}$$

$$\frac{\mathrm{d}Ca_{SR}}{\mathrm{d}time} = \frac{Ca_{sr_{bufsr}} \cdot V_c}{V_{sr}} \cdot \left(I_{up} - \left(I_{rel} + I_{leak}\right)\right) \tag{S99}$$

1.18 Ca^{2+} pump current, I_{pCa}

$$I_{p_{Ca}} = \frac{g_{pCa} \cdot Ca_i}{Ca_i + K_{pCa}} \tag{S100}$$

1.19 Reversal potentials

$$E_{Na} = \frac{R \cdot T}{F} \cdot \ln \frac{Na_o}{Na_i} \tag{S101}$$

$$E_K = \frac{R \cdot T}{F} \cdot \ln \frac{K_o}{K_i} \tag{S102}$$

$$E_{Ks} = \frac{R \cdot T}{F} \cdot \ln \frac{K_o + P_{kna} \cdot Na_o}{K_i + P_{kna} \cdot Na_i} \tag{S103}$$

$$E_{Ca} = \frac{0.5 \cdot R \cdot T}{F} \cdot \ln \frac{Ca_o}{Ca_i} \tag{S104}$$

$$E_f = -0.017\ (V) \tag{S105}$$

1.20 Sodium dynamics

$$\frac{\mathrm{d}Na_i}{\mathrm{d}time} = -C_m \cdot \frac{I_{Na} + I_{NaL} + I_{fNa} + I_{bNa} + 3I_{NaK} + 3I_{NaCa}}{F \cdot V_c} \tag{S106}$$

1.21 Background currents

$$I_{bNa} = g_{bNa} \cdot \left(V - E_{Na}\right) \tag{S107}$$

$$I_{bCa} = g_{bCa} \cdot \left(V - E_{Ca}\right) \tag{S108}$$

2 Delayed afterdepolarizations

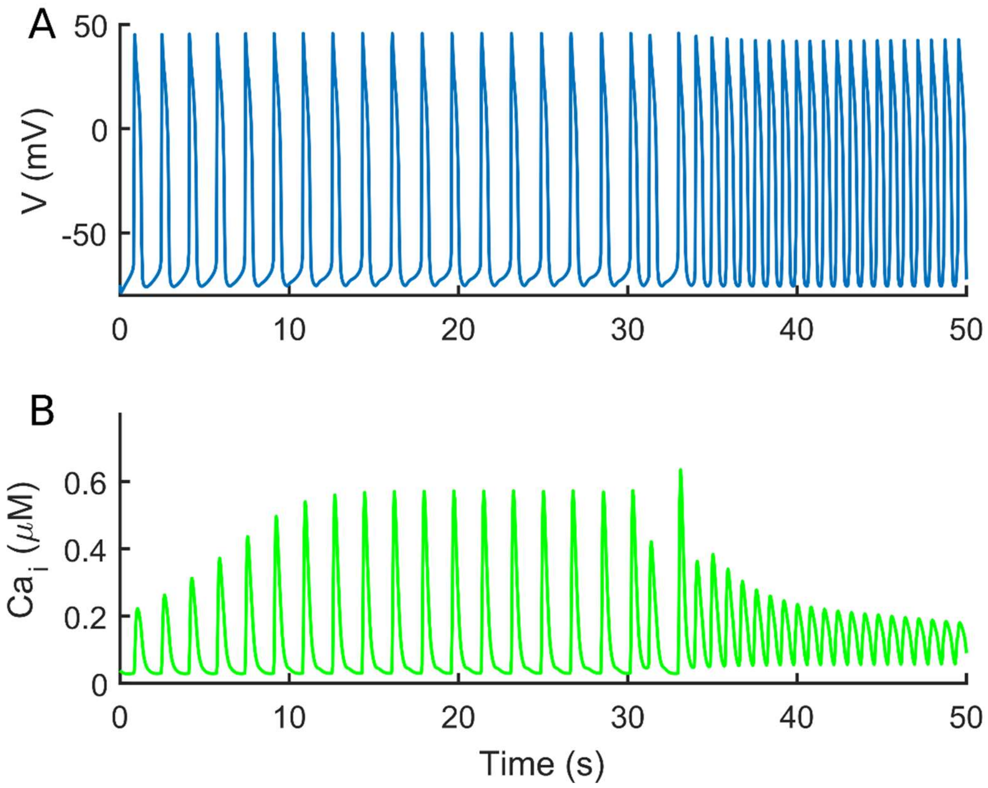

Supplementary Figure 1. Time-courses of the membrane potential (A) and cytosolic Ca^{2+} (B) of the Paci2018 in conditions of hypercalcemia. The DAD-like abnormalities at second 31 cause a transition to a higher rate of Ca^{2+} transients and APs.

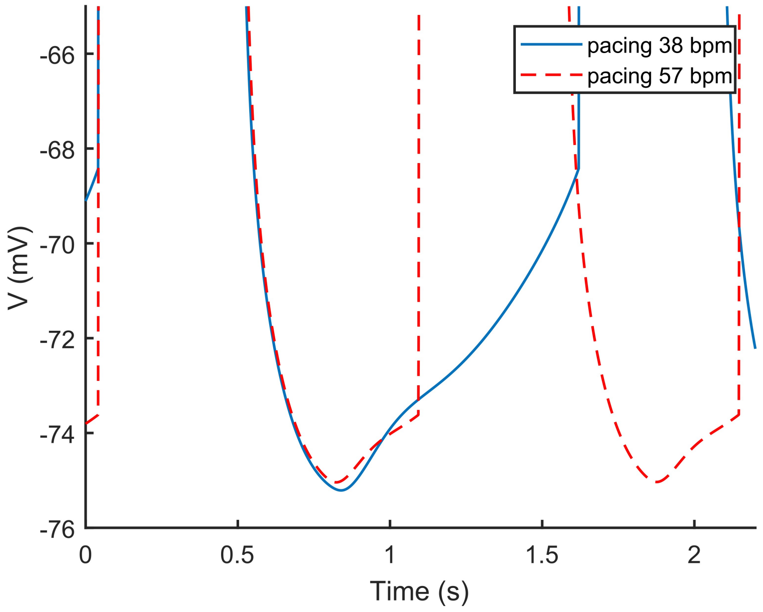

Supplementary Figure 2. DADs in APs obtained by pacing the Paci2018 model at different frequencies in hypercalcemia conditions. On one hand, the blue solid trace shows APs paced at 38 bpm, corresponding to the spontaneous AP rate. On the other hand, the red dashed trace shows APs paced at 57 bpm, i.e. we increased the pacing rate by 50%. Also at 57 bpm, despite the shorter diastolic phase, we obtained a fully developed DAD.

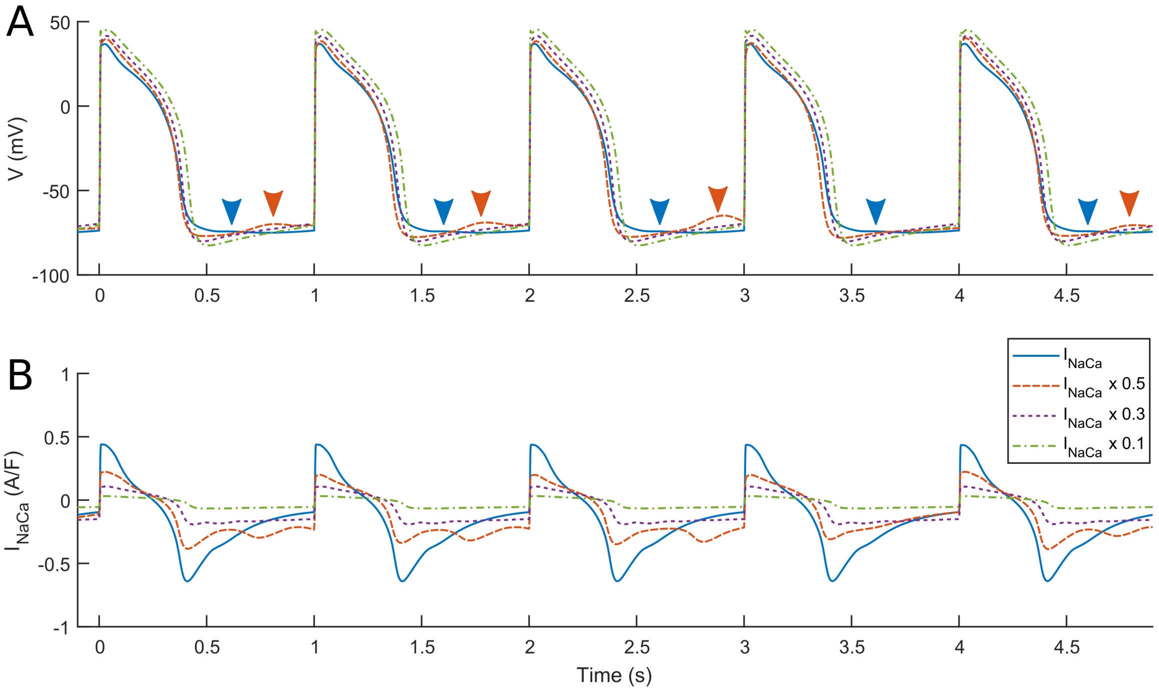

Supplementary Figure 3. Effect of I_{NaCa} block on DADs. (A) APs traces. (B) I_{NaCa} traces. DADs were obtained as in Figure 7 at control extracellular Ca^{2+} concentration, by shifting $RyR_{o,half}$ and $RyR_{c,half}$

by -0.002 and 0.002 mM respectively, doubling RyR_o time constant and reducing to half of its nominal value RyR_c time constant. The Paci2018 was paced at 1 Hz (stimulus amplitude 750 pA). The solid blue AP trace (no I_{NaCa} block) shows small DADs. The 50% I_{NaCa} block (dashed orange line) is not enough to cancel DADs: in fact, the inward I_{NaCa} component is still strong enough and further enhanced by the cytosolic Ca^{2+} accumulation, due to the very same I_{NaCa} block. This results in even larger DADs than in the no block case. DADs did not occur by blocking 70% (dashed purple) and 90% (dashed green) I_{NaCa}.

3 Early afterdepolarizations

We tested the capability of the Paci2018 model to generate early afterdepolarizations (EADs) by blocking I_{Kr} by 90% from steady state conditions. I_{Kr} block was tested i) on the original Paci2018, whose parameter set was obtained by means of the optimization presented in the main manuscript, and ii) in three additional hiPSC-CMs models generated by modulating the maximum conductances/currents of the Paci2018 model, as done in (Paci et al. 2016). In detail, the maximum conductances/currents of I_{Na}, I_{NaL}, I_{CaL}, I_f, I_{to}, I_{Kr}, I_{Ks}, I_{K1}, I_{NaCa}, I_{NaK} and I_{pCa} were rescaled according to coefficients included in the range [0.5, 2]. The three sets of coefficients were chosen among those that generated repolarization abnormalities (EADs or repolarization failure) in (Paci et al. 2016) and they are presented in the Supplementary Table 1.

The effect of the 90% I_{Kr} block on the four hiPSC-CM models is shown in Supplementary Figure 4. The Paci2018 model (blue) did not produce repolarization abnormalities. Conversely, the models produced by means of the coefficient sets 2 (in green) and 3 (in red) showed different patterns of EADs. Finally, set 4 (in magenta) produced a model which failed to repolarize without producing EADs.

Supplementary Table 1. Sets of coefficients used to rescale the maximum conductances and currents in the Paci2018 model. The first coefficient set corresponds to the baseline Paci2018 model. The other coefficient sets were taken from (Paci et al. 2016), in order to represent repolarization abnormalities. Column AB reports the repolarization abnormalities observed as consequence of a 90% block of I_{Kr}: for sets 2 and 3 we observed EADs, while for set 4 we observed repolarization failure (RF) without EADs.

ID	G_{Na}	G_f	G_{CaL}	G_{to}	G_{Ks}	G_{Kr}	G_{K1}	I_{NaCa}	I_{NaK}	G_{pCa}	G_{NaL}	AB
1	1.0000	1.0000	1.0000	1.0000	1.0000	1.0000	1.0000	1.0000	1.0000	1.0000	1.0000	NO
2	1.6890	1.9912	1.6558	0.8900	1.1459	1.8201	1.0149	1.0927	1.6938	0.5574	1.3234	EAD
3	1.3978	1.8679	1.1943	1.2455	1.9848	1.6000	0.5817	1.2807	1.5267	1.0590	0.7438	EAD
4	1.0621	1.8146	1.6017	1.8869	1.9849	1.6180	0.6656	1.3178	1.3769	1.7040	1.2419	RF

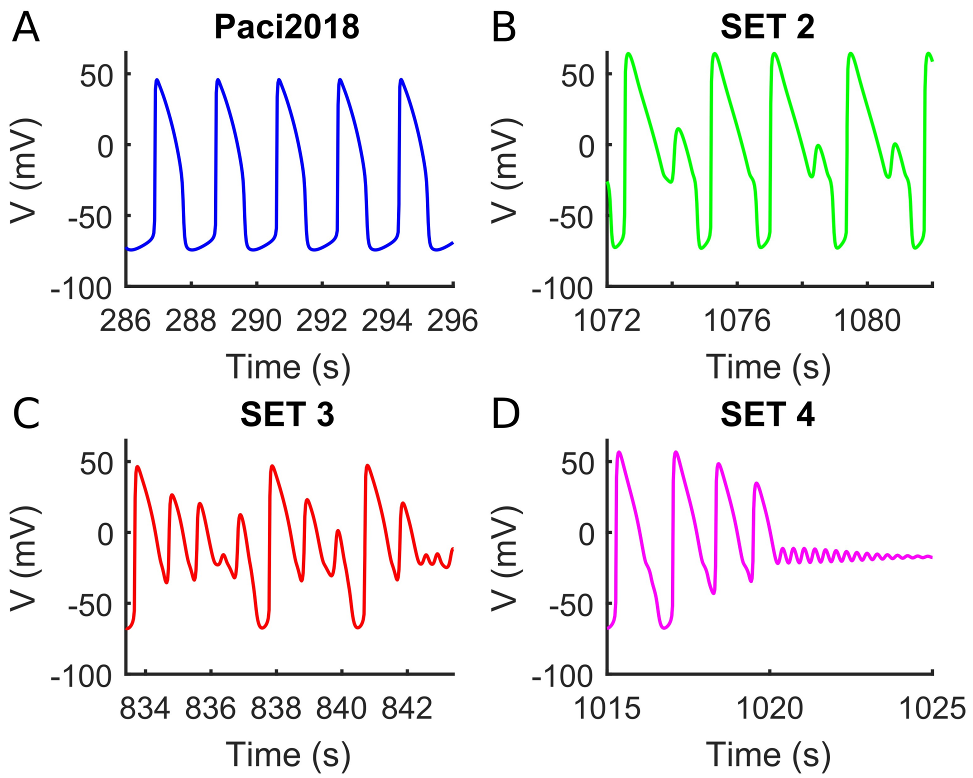

Supplementary Figure 4. Effects of 90% I_{Kr} block on the Paci2018 model (blue) and the three hiPSC-CM models derived from it (green, red and magenta).

4 Alternans-like patterns

We tested the capability of the Paci2018 model to produce alternans in two different conditions.

The first test consisted in pacing at 120 bpm (stimulus amplitude 900 pA, pulse duration 5 ms) the Paci2018. We observed that an alternans-like pattern emerged, as shown in Supplementary Figure 5, panel A

The second test aimed to assess alternans in ischemia-like conditions. We modified the Paci2018 model similarly as in (Hopenfeld 2006), to replicate ischemic conditions. In particular, i) K_o = 12 mM to simulate hyperkalemia, ii) I_{to} maximum conductance was multiplied by 2.3 and iii) I_{CaL} maximum conductance was reduced to 40% of its nominal value. The Paci2018 model was then paced at very high frequency (200 bpm, stimulus amplitude 900 pA, and pulse duration 5 ms). We observed that i) the maximum diastolic potential was strongly depolarized, as commonly observed in ischemia conditions, and that ii) 2:1 alternans appeared, as shown in the Supplementary Figure 5, panel B.

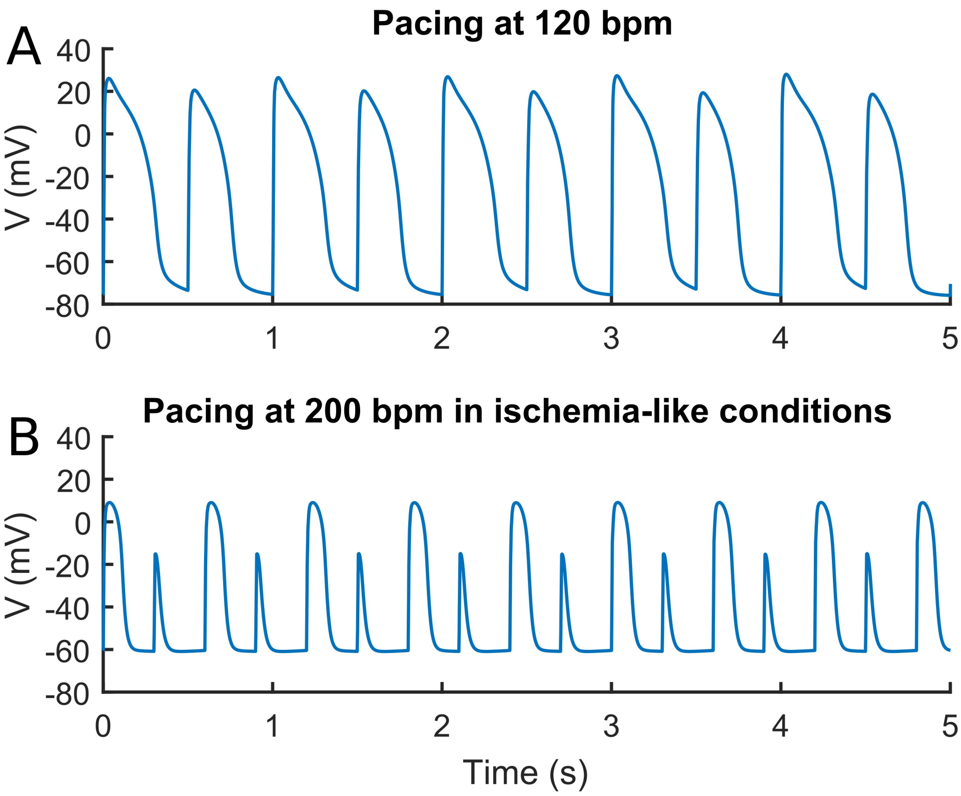

Supplementary Figure 5. Alternans simulations with the Paci2018 model. (A) Alternans-like patterns emerging when stimulating the Paci2018 model at 120 bpm (2 Hz). (B) 2:1 alternans emerging when pacing the Paci2018 at 200 bpm (3.33 Hz) in ischemia-like conditions.

5 References

Hopenfeld, Bruce. 2006. "Mechanism for Action Potential Alternans: The Interplay between L-Type Calcium Current and Transient Outward Current." *Heart Rhythm* 3 (3):345–52. doi: 10.1016/j.hrthm.2005.11.016.

Paci, Michelangelo, Elisa Passini, Stefano Severi, Jari Hyttinen, and Blanca Rodriguez. 2016. "A Population of in Silico Models to Face the Variability of Human Induced Pluripotent Stem Cell-Derived Cardiomyocytes: The HERG Block Case Study." In *Computing in Cardiology*, 1189–92. doi: 10.23919/CIC.2016.7868961.
